# Progerin in muscle leads to thermogenic and metabolic defects via impaired calcium homeostasis

**DOI:** 10.1111/acel.13090

**Published:** 2019-12-12

**Authors:** Wan‐Ping Wang, Jing‐Ya Wang, Wen‐Hsin Lin, Cheng‐Heng Kao, Ming‐Chun Hung, Yuan‐Chi Teng, Ting‐Fen Tsai, Ya‐Hui Chi

**Affiliations:** ^1^ Institute of Biotechnology and Pharmaceutical Research National Health Research Institutes Zhunan Taiwan; ^2^ Center of General Education Chang Gung University Taoyuan Taiwan; ^3^ Department of Life Sciences and Institute of Genome Sciences National Yang‐Ming University Taipei Taiwan; ^4^ Graduate Institute of Biomedical Sciences China Medical University Taichung Taiwan

**Keywords:** aging, calcium homeostasis, lamin A, muscular dystrophy, progeria

## Abstract

Mutations in lamin A (*LMNA*) are responsible for a variety of human dystrophic and metabolic diseases. Here, we created a mouse model in which progerin, the lamin A mutant protein that causes Hutchinson–Gilford progeria syndrome (HGPS), can be inducibly overexpressed. Muscle‐specific overexpression of progerin was sufficient to induce muscular dystrophy and alter whole‐body energy expenditure, leading to premature death. Intriguingly, sarcolipin (Sln), an endoplasmic reticulum (ER)‐associated protein involved in heat production, is upregulated in progerin‐expressing and *Lmna* knockout (*Lmna* ^−/−^) skeletal muscle. The depletion of *Sln* accelerated the early death of *Lmna* ^−/−^ mice. An examination at the molecular level revealed that progerin recruits Sln and Calnexin to the nuclear periphery. Furthermore, progerin‐expressing myoblasts presented enhanced store‐operated Ca^2+^ entry, as well as increased co‐localization of STIM1 and ORAI1. These findings suggest that progerin dysregulates calcium homeostasis through an interaction with a subset of ER‐associated proteins, resulting in thermogenic and metabolic abnormalities.

## Graphical abstract

A model for progerin in calcium homeostasis and thermogenesis. In the molecular level, progerin can recruits a subset of endoplasmic reticulum (ER) proteins including Sln and Calnexin, but not SERCA2 or Calreticulin to the nuclear periphery, and may thus induce ER stress and enhance store‐operated calcium entry. The disturbed calcium homeostasis in progeric muscle may trigger transcriptional activation of Sln and ER‐stress associated genes and alter muscle‐based thermogenesis, leading to premature death of the animals. Cyt., cytosolic.

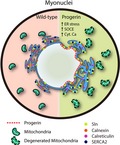

## 1 INTRODUCTION

The nuclear lamina encoded by *LMNA*, *LMNB1,* and *LMNB2* is a meshwork of type V intermediate filament proteins underlying the inner nuclear membrane (INM). Mutations in nuclear lamin genes have been linked to human diseases with phenotypes ranging from cardiac and skeletal myopathies, lipodystrophy, peripheral neuropathy, and premature aging, such as Hutchinson–Gilford progeria syndrome (HGPS), collectively termed laminopathies (Chi, Chen, & Jeang, 2009; Winder, 2006). These effects have been attributed to the intrinsic molecular function of nuclear lamins, including structure and assembly of the nuclear lamina, gene expression, DNA damage repair, cell cycle control, chromatin organization, and signaling and function of the endoplasmic reticulum (ER; Winder, 2006). However, there remain a number of questions regarding the means by which mutations in the ubiquitously expressed lamin A give rise to clinically unrelated pathologies affecting specific tissues (Chi et al., 2009; Janin, Bauer, Ratti, Millat, & Mejat, 2017).

The nuclear envelope (NE) is composed of two layered membranes contiguous with the ER; therefore, distortions of the nuclear lamina caused by *LMNA* mutations are expected to affect both the structure and function of the ER. The ER is essential to a variety of cellular functions, including protein synthesis and folding, lipid synthesis, and calcium (Ca^2+^) sequestration and release (Phillips & Voeltz, 2016). Researchers, including our team, have reported that the accumulation of progerin and an inner nuclear membrane protein SUN1 (SAD1‐UNC84 domain protein 1) contributes to the dystrophic appearance of laminopathies (Chen et al., 2012; McClintock, Gordon, & Djabali, 2006). The dysregulated interactions between progerin (the lamin A mutant protein that causes HGPS) and SUN1 recruit excess ER membrane to the nuclear periphery (Chen et al., 2014). In a *Drosophila melanogaster* model, it was shown that the expression of the human disease‐causing *Lamin C* mutant protein in the heart can lead to the cytoplasmic aggregation of LamC and Otefin (a homolog of human Emerin) as well as the disruption of myofibrils (Bhide et al., 2018). A recent study reported that ER stress and the unfolded protein response (UPR) were activated in cultured cells derived from HGPS patients and in some organs of a HGPS mouse model (Hamczyk et al., 2019).

ER stress can be elicited by disturbances in cellular energy levels including the redox state or Ca^2+^ concentrations, leading to accumulation and aggregation of unfolded proteins (Ron & Walter, 2007). In this study, we developed a mouse model in which human progerin is conditionally overexpressed in muscle and then explored the physiological consequence of this disease‐causing lamin A mutant and the association with sarcoplasmic reticulum (SR)/ER function. Our results demonstrate that the conditional overexpression of progerin in muscle is sufficient to cause premature death and dysregulate the expression of thermogenesis‐associated genes. Our analysis at the molecular level revealed that the expression of progerin disrupted calcium homeostasis, thereby disturbing whole‐body energy expenditure.

## 2 RESULTS

### 2.1 Upregulation of *Sln* ameliorates early death of *Lmna* ^−/−^ mice

**Figure 1:**
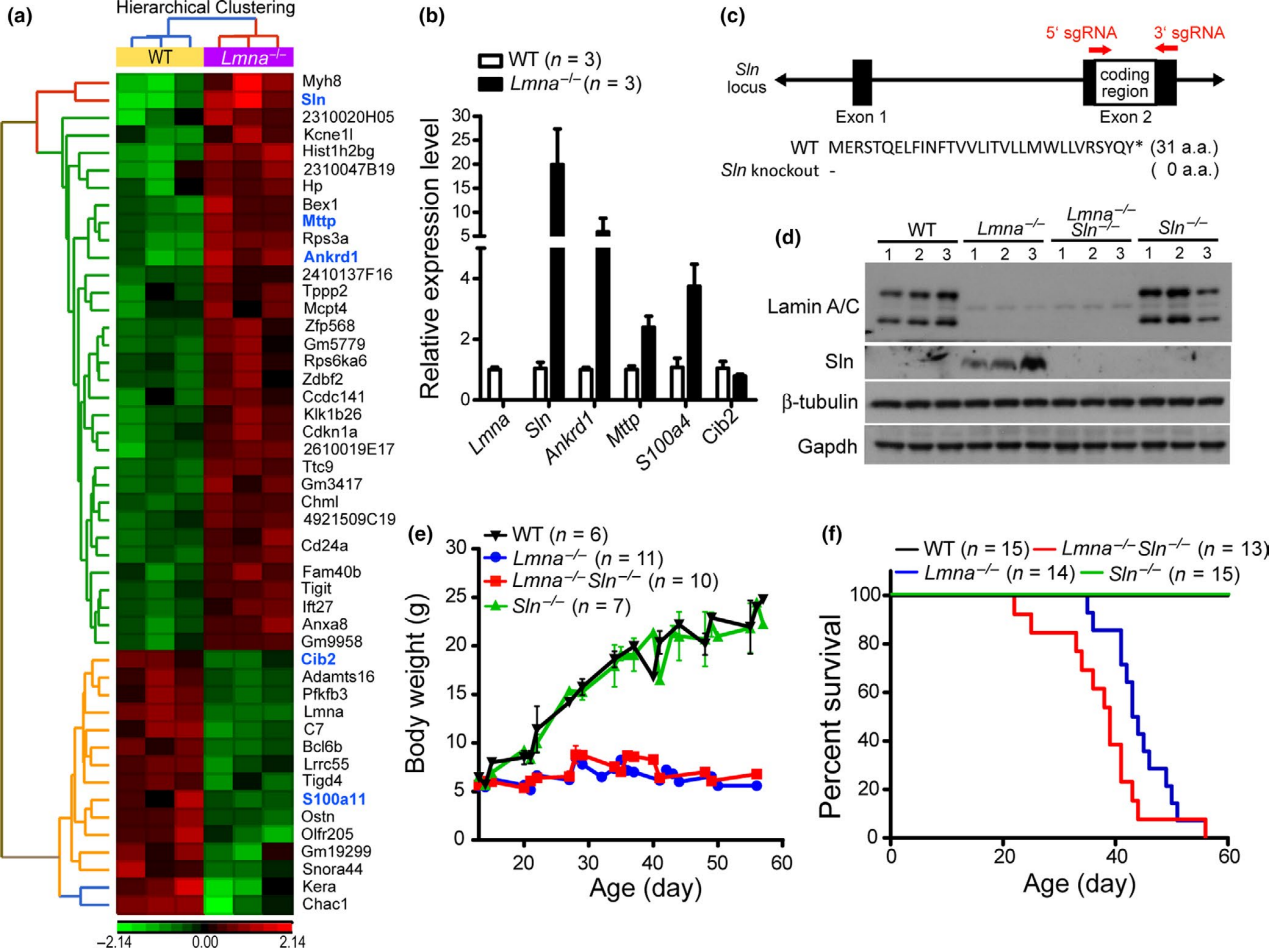
*Sln* expression extends the lifespan of *Lmna* ^−/−^ mice. (a) Microarray analysis for the differentially expressed genes between 30‐day‐old male wild‐type (WT; *n* = 3) and *Lmna* ^−/−^ (*n* = 3) femoris muscle. The upregulated genes are shown in red, and the down‐regulated genes are shown in green. Genes that are associated with endoplasmic reticulum (ER) function are highlighted in blue letters. (b) Relative expression levels of the indicated genes in 30‐day‐old male *Lmna* ^−/−^ skeletal muscle compared to the WT cohorts by qRT‐PCR. (c) Schematic representation of the construction strategy for *Sln* knockout mice using the CRISPR/Cas9 techniques. The *Sln* knockout mice used in this study lost the entire coding region of *Sln*. (d) Western blotting for verification of lamin A/C and Sln protein expression in the soleus muscle of 30‐day‐old male WT, *Lmna* ^−/−^, *Lmna* ^−/−^ *Sln* ^−/−^, and *Sln* ^−/−^ mice. For each genotype, the expression profiles from three mice were shown. Immunoblots of β‐tubulin and Gapdh are shown as loading controls. (e) Growth curve in body weight of WT, *Lmna* ^−/−^, *Lmna* ^−/−^ *Sln* ^−/−^, and *Sln* ^−/−^ mice (male and female). (f) Kaplan–Meier survival curve of WT, *Lmna* ^−/−^, *Lmna* ^−/−^ *Sln* ^−/−^, and *Sln* ^−/−^ mice (male and female). Comparing *Lmna* ^−/−^ and *Lmna* ^−/−^ *Sln* ^−/−^ mice, *p* = .0579 using log‐rank (Mantel–Cox) test, and *p* = .0111 using Gehan–Breslow–Wilcoxon test. All values are mean ± *SEM*

The physiological function of lamin A in vivo was first revealed in *Lmna* knockout (*Lmna* ^−/−^) mice, which presented a mosaic phenotype of laminopathies, including muscular dystrophy and sciatic neuropathy (Sullivan et al., 1999). *Lmna* ^−/−^ mice actually express a truncated 54 kDa lamin A dominant‐negative mutant, which can be farnesylated (Jahn et al., 2012). Here, we preserved the original nomenclature (i.e., *Lmna* ^−/−^) of this mouse line. Our objective in this study was to elucidate the defects of *Lmna* mutation‐elicited muscle dysfunction. Using the Affymetrix mouse gene 2.0ST array, we identified perturbed genes in *Lmna* ^−/−^ skeletal muscle and discovered the upregulation of *Sln* (Figure 1a). Several other genes associated with ER function (blue letters) were also differentially expressed, including *Mttp*, *Ankrd1*, *Cib2*, and *S100a4* (1.9‐fold increase in microarray). Their expression levels were further verified by qRT‐PCR (Figure 1b).

Researchers have observed increased *Sln* expression levels in various forms of muscular dystrophy (Bal et al., 2012; Fajardo et al., 2018, 2017; Pant, Bal, & Periasamy, 2016). To characterize the physiological role of *Sln* upregulation in *Lmna* ^−/−^ mice, we created *Sln* knockout (*Sln* ^−/−^) mice (Figure 1c) using the CRISPR/Cas9‐mediated genome engineering technique (Zhou et al., 2014). Western blot analysis confirmed that the protein level of Sln was higher in *Lmna* ^−/−^ skeletal muscle than in the wild‐type (WT) cohorts and was absent in *Lmna* ^−/−^ *Sln* ^−/−^ mice (Figure 1d). We observed similarities in body weight between *Lmna* ^−/−^ *Sln* ^−/−^ and *Lmna* ^−/−^ mice (Figure 1e); however, the medial lifespan of *Lmna* ^−/−^ *Sln* ^−/−^ mice was lower due to a significant increase in the incidence of early death (*p* < .0111, Gehan–Breslow–Wilcoxon test, Figure 1f). These results suggest that the upregulation of *Sln* can attenuate the premature death of *Lmna* ^−/−^ mice.

### 2.2 Conditional overexpression of human progerin in muscle induces premature death in mice

**Figure 2:**
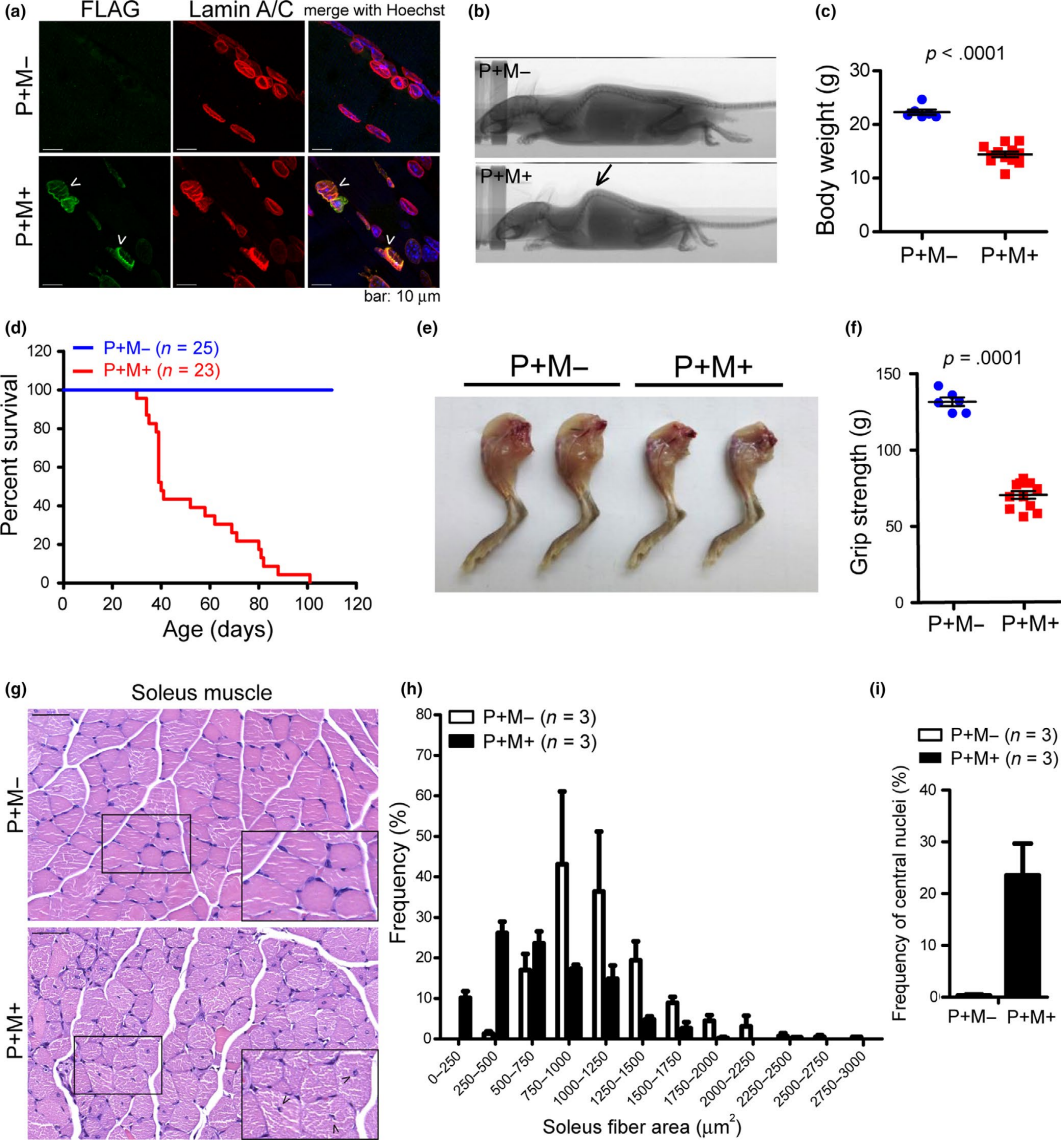
Expression of human progerin in mouse muscle leads to muscular dystrophy and early death. (a) Immunofluorescence staining for the expression of FLAG‐tagged progerin (green) and lamin A/C (red) in the skeletal muscle of 2‐month‐old male P+M− and P+M+ mice. (b) X‐ray images of 2‐month‐old P+M− and P+M+ mice (male). The P+M+ mice appeared kyphotic (indicated by an arrow). (c) Body weight of 2‐month‐old male P+M− (*n* = 6) and P+M+ mice (*n* = 12). (d) Kaplan–Meier survival curve of P+M− and P+M+ mice (male and female). *p* = .0017 using log‐rank (Mantel–Cox) test. (e) Pictures of the hindlimbs dissected from 2‐month‐old P+M− and P+M+ mice (male). (f) Grip strength of P+M− (*n* = 6) and P+M+ (*n* = 12) mice (male). The value was obtained from an average of 5 tests for each mouse. Considering body weight as a covariate in this measurement, two‐way ANOVA was used to compute the p value. (g) Representative hematoxylin and eosin (H&E) staining paraffin sections of soleus muscle from 2‐month‐old P+M− and P+M+ mice (male). Myocytes with central nuclei are denoted by arrow heads. Bars: 50 μm. (h) Frequency histograms showing the distribution of myofiber cross‐sectional area in P+M− and P+M+ soleus muscle. (i) Quantification of the central nuclei frequency in the soleus muscle of P+M− and P+M+ mice. All values are mean ± *SEM*

Sarcolipin (SLN) is an ER‐associated protein important to muscle‐based thermogenesis in mammals (Bal et al., 2012). Skeletal muscle constitutes ∼40% of the body mass and operates as a thermogenic, metabolic, and endocrine organ (Pant et al., 2016; Rowland, Bal, Kozak, & Periasamy, 2015). Metabolic phenotypes are characteristic of HGPS patients (Charar & Gruenbaum, 2017; Liao et al., 2016; Ramos et al., 2012). Thus, we sought to determine whether dysregulated lamin A expression in muscle affects energy expenditure throughout the entire body. This was achieved by creating a knock‐in mouse model, which conditionally overexpresses human progerin (tagged with FLAG) in muscle tissue driven by the CAG promoter and MCK (muscle creatine kinase)‐Cre (Figure S1a,b). It has been reported that the earliest MCK‐Cre activation is detectable in multinucleate myotubes/myofibers (Bi et al., 2016). Western blot analysis indicated that the expression of FLAG‐progerin is specifically induced in the cardiac and skeletal muscle of *CAG‐Progerin^+^; MCK‐Cre^+^* (i.e., P+M+) mice, but not in *CAG‐Progerin^+^* only (i.e., P+M−) littermates (Figure S1c). A mild expression of FLAG‐progerin was observed in the skin and brown adipose tissue (BAT) of P+M+ mice, perhaps due to the intermingled smooth muscle layer in the tissues. We observed that the expression of endogenous *Lmna* in the muscle of P+M+ mice was slightly elevated at the transcription and protein levels, perhaps in compensation for the hindered protein function (Figure S1d,e). Immunofluorescence staining revealed that the nuclear morphology of P+M+ skeletal muscle became ruffled under the expression of FLAG‐progerin (denoted by arrow heads, Figure 2a), similar to HGPS skin fibroblasts (Goldman et al., 2004). Approximately 30%–45% of the cells in the heart and skeletal muscle of 2‐month‐old P+M+ mice stained positive for FLAG‐progerin (32.0% in heart, 55/172; 43.0% in gastrocnemius muscle, 67/156).

Adult (i.e., 2‐month‐old) P+M+ mice appeared smaller than their P+M− cohorts and presented significant kyphosis (Figure 2b). The growth curve of P+M+ mice began deviating from that of P+M− mice in the third week after birth (Figure S1f). Body weight of 2‐month‐old male P+M+ mice (14.4 ± 0.51 g, *n* = 12) was approximately 35% lower than that of the P+M− cohort (22.3 ± 0.50 g, *n* = 6, Figyre 2c). As with the *Lmna* ^−/−^ mice (Chen et al., 2012), the P+M+ mice died prematurely, resulting in a medial lifespan of 40.0 days (Figure 2d). A reduction in muscle mass (Figure 2e) manifests as a 50% reduction in the grip strength of P+M+ mice compared to the P+M− littermates (Figure 2f). A histological examination of the soleus muscle revealed a reduction in muscle cross‐sectional area (CSA) and an increase in the prevalence of central nuclei in the myofibrils of P+M+ mice (Figure 2g–i). Electrocardiography (ECG) results revealed that P+M+ mice tend to have a lower heart rate (*p* = .0627) and higher QT interval (*p* = .0440, Figure S1g), which are suggestive of weaker cardiac function. The significant increase in serum levels of creatine phosphokinase (CPK) is an indication of dystrophy in the heart and muscle of P+M+ mice (Figure S1h). Despite reports that the chronic expression of Cre recombinase in the heart causes cardiac toxicity in adult mice, we did not observe any difference between *MCK‐Cre^+^* (i.e., P−M+) mice and wild‐type (WT) mice in terms of behavior or ECG profiles (Figure S2). These results demonstrate that progerin expression in muscle is sufficient to induce progeric phenotypes and provoke premature death in mice.

### 2.3 Progerin in muscle impairs heat production and affects whole‐body energy expenditure

**Figure 3:**
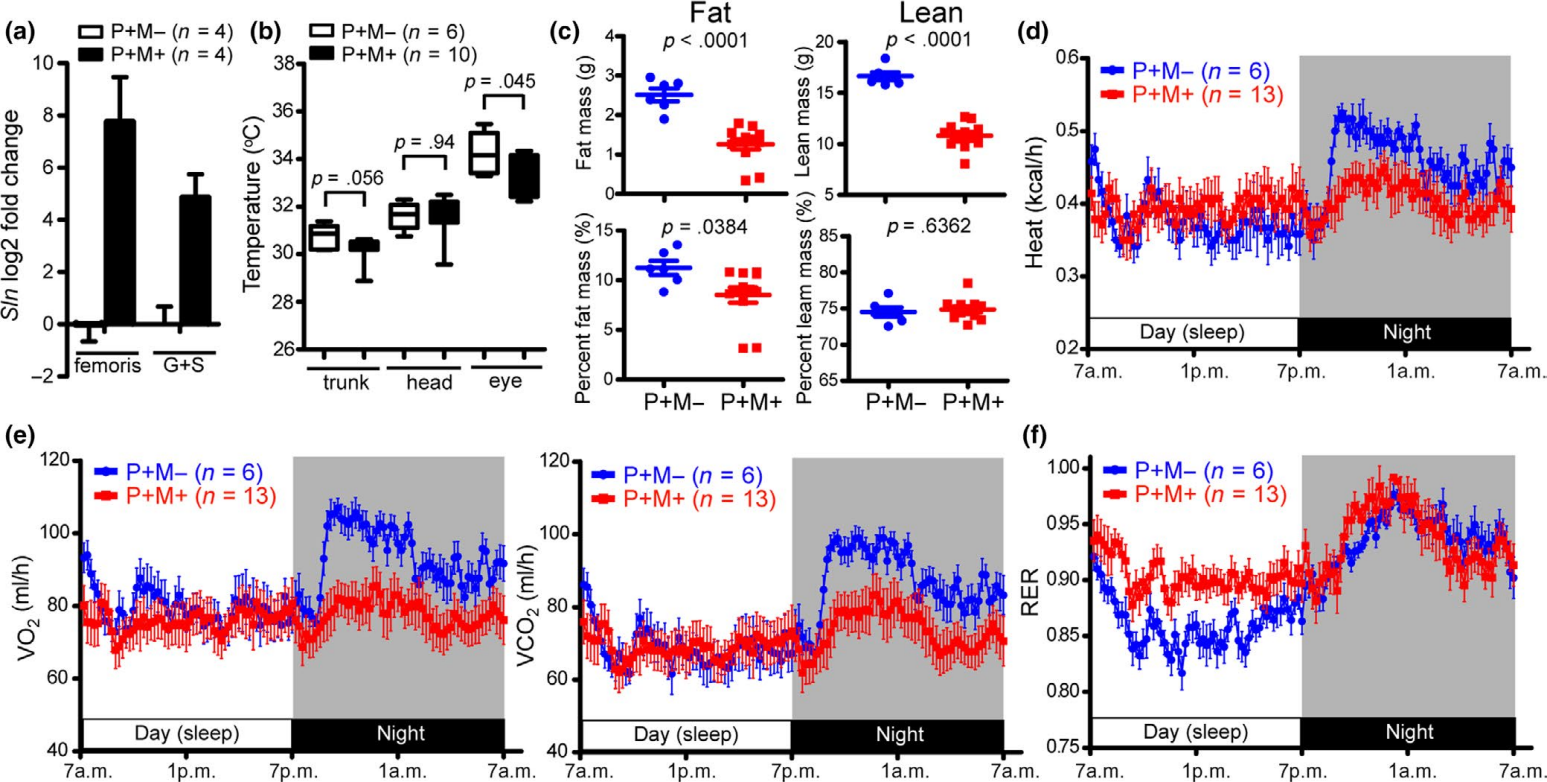
Progerin overexpression in muscle dysregulates thermogenesis and metabolism. (a) The expression levels of *Sln* mRNA in 2‐month‐old P+M− and P+M+ femoris and gastrocnemius+soleus (G+S) muscle (male) was quantified using qRT‐PCR. (b) Body surface temperatures in the trunk, head, and eye of 2‐month‐old P+M− and P+M+ mice (male). (c) Quantification of fat mass and lean mass in 2‐month‐old male P+M− (*n* = 6) and P+M+ (*n* = 12) mice measured by a high‐resolution X‐ray micro‐CT system. (d–f) Kinetic data for heat production (d), oxygen consumption (VO_2_, e), carbon dioxide production (VCO_2_, e), and respiratory exchange ratio (RER, f) in P+M− and P+M+ mice (male). Results are averaged values from 2‐day monitoring using CLAMPS. All values are mean ± *SEM*

Consistent with the observations of *Lmna* ^−/−^ mice, the overexpression of progerin in muscle proved sufficient for the upregulation of *Sln* (Figure 3a), which is suggestive of aberrant thermogenesis. The surface temperature of the eye and trunk was lower in P+M+ mice than in P+M− mice (eye: 34.3 ± 0.349°C in P+M− vs. 33.3 ± 0.255°C in P+M+, *p* = .0432; trunk: 30.8 ± 0.203°C in P+M− vs. 30.2 ± 0.162°C in P+M+, *p* = .0565; Figure 3b). We opted not to obtain rectal temperature measurements as this procedure may increase stress levels in P+M+ mice that are physiologically vulnerable. Intriguingly, the net volume and ratio of fat mass was significantly lower in P+M+ mice (8.53 ± 0.772% fat, 74.9 ± 0.427% lean) than in the P+M− cohort (11.3 ± 0.706% fat, 74.5 ± 0.650% lean; Figure 3c). Likewise, the cell volume of white adipose tissue (WAT) was considerably lower in P+M+ mice than in P+M− mice (Figure S3a), whereas the histology of brown adipose tissue (BAT) was unaffected (Figure S3b). We observed an overall reduction in muscle volume in P+M+ mice (Figure 2e); however, the percentage of lean mass was unaffected, perhaps due to the fact that lean mass accounts for ~ 75% of the body weight of mice.

Progerin is not much expressed in the adipose tissues of P+M+ mice (Figures S1c and S3c); therefore, the reduced fat mass in P+M+ mice implies that the progerin‐induced muscle loss may affect metabolism throughout the entire body. We therefore compared behavior and energy metabolism in P+M+ and P+M− mice using homecage activity analysis and CLAMS‐HC (Comprehensive Lab Animal Monitoring System for HOME CAGES). We did not observe a significant difference between P+M− and P+M+ mice in terms of daytime activity (Figure S3d). Conversely, we observed significant differences in night activity, wherein the P+M+ mice presented significantly reductions in the frequencies of drinking, hanging, rearing, and walking compare to the P+M− cohorts. Interestingly, the frequency of twitching was significantly higher in P+M+ mice (Figure S3e). No difference was observed between P+M+ mice and P+M− mice in terms of heat production during the day; however, heat production in P+M+ mice was lower at night (Figure 3d). In the P+M− mice, the whole‐body consumption of oxygen (VO_2_) and carbon dioxide production (VCO_2_) per unit activity (ml/h) were higher during the night than during the daytime (Figure 3e), which is suggestive of increased activity at night. VO_2_ and VCO_2_ were both lower in P+M+ mice than in P+M− mice at night. These results correlate with the overall lower activity levels observed in P+M+ mice during the night time (Figure S3e). The P+M− mice presented a respiratory exchange ratio (RER, Figure 3f) of <0.85 during the day and >0.85 during the night, whereas the RER of P+M+ mice was always >0.85. Collectively, these results show that P+M+ mice have a lower body temperature, are less active at night, and are prone to use carbohydrates instead of fat as a fuel source even at rest (Subkhangulova et al., 2018).

### 2.4 Progerin interacts with and recruits ER‐associated proteins to the nuclear periphery

**Figure 4:**
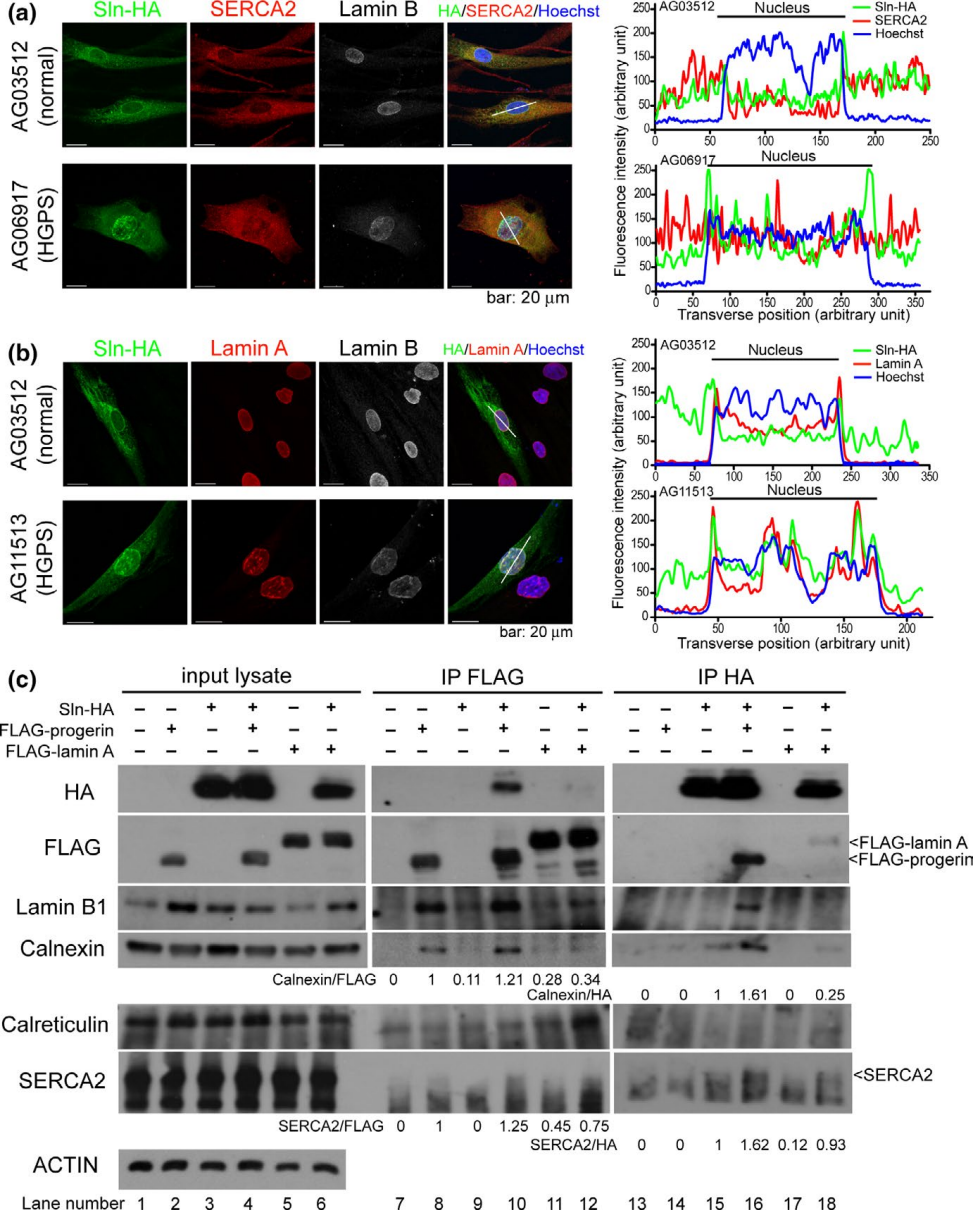
The interaction of nuclear lamins and endoplasmic reticulum (ER) proteins. (a and b) Normal and HGPS skin fibroblasts were transfected with a Sln expression vector tagged with HA (i.e., Sln‐HA). Cells were fixed after 96 hr of transfection and immunofluorescent stained to visualize HA (green), cell endogenous SERCA2 or lamin A (red), and lamin B (gray). The nuclei were counterstained using Hoechst 33,342 (blue). Transverse intensity line scans along the white lines of the corresponding cell images are presented on the right. (c) HEK293TN cells were co‐transfected with Sln‐HA, FLAG‐progerin and/or FLAG‐lamin A. Cells transfected with (+) or without (−) the indicated expression plasmids are noted at the top of the Western blot profiles. Cell lysates were immunoprecipitated using mouse anti‐HA and mouse anti‐FLAG agarose, respectively. The cell lysate input and co‐IP products were analyzed by SDS‐PAGE followed by immunoblotting with the indicated antibodies. The signals of the co‐immunoprecipitated Calnexin and SERCA2 were quantified and shown below the immunostaining profiles

Sln has been shown to promote mitochondrial biogenesis and oxidative metabolism in skeletal muscle (Maurya et al., 2018). Sln is >10 fold overexpressed in the skeletal muscle of *Lmna* ^−/−^ and P+M+ mice (Figures 1b and 3a); however, its protective effects (in terms of the overall lifespan of *Lmna* ^−/−^ mice) appear to be limited (Figure 1f). Sln resides in the ER; therefore, we sought to determine whether progerin plays a role on Sln function. We first examined the localization of Sln (tagged with HA) in normal and HGPS skin fibroblasts. Unlike SERCA2, which is homogenously distributed throughout the ER network, Sln tends to co‐localize with lamin B and lamin A at the nuclear periphery in normal skin fibroblasts (Figure 4a,b). Moreover, the co‐localization of Sln with the nuclear lamina was more pronounced in HGPS skin fibroblasts than in normal cells (Figure 4a,b).

We then determined whether Sln is associated with nuclear lamins through co‐immunoprecipitation. Sln is embedded in the ER membrane (which is difficult to extract); therefore, we used 2 mM dithiobis (succinimidyl propionate) to crosslink interacting proteins before lysing the cells (Lynes et al., 2013). The reciprocal immunoprecipitation profiles of progerin/lamin A (tagged with FLAG) and Sln (tagged with HA) indicate that Sln does indeed interact with lamin A, and its association with progerin is significantly higher (Figure 4c, compare lanes 9–12 and 15–18). Furthermore, Sln pulled down lamin B1 along with progerin but not lamin A (Figure 4c, lanes 15–18). To determine whether progerin/lamin A is associated with other ER proteins, we looked for the existence of Calnexin, Calreticulin, and SERCA2 in the immunoprecipitated products. Our results revealed that Calreticulin is not associated with lamin A, progerin, or Sln, whereas Calnexin interacted with lamin A, progerin, and Sln (Figure 4c, compare lanes 8, 11, and 15). Sln interacted with cell endogenous SERCA2, and the expression of progerin enhanced the interaction between SERCA2 and Sln (Figure 4c, compare lanes 15–16 with 13–14 and Figure S4). These results suggest that progerin is associated with a subset of ER proteins, including Sln and Calnexin.

### 2.5 Enhanced store‐operated calcium entry (SOCE) in progerin‐expressing myoblasts

**Figure 5:**
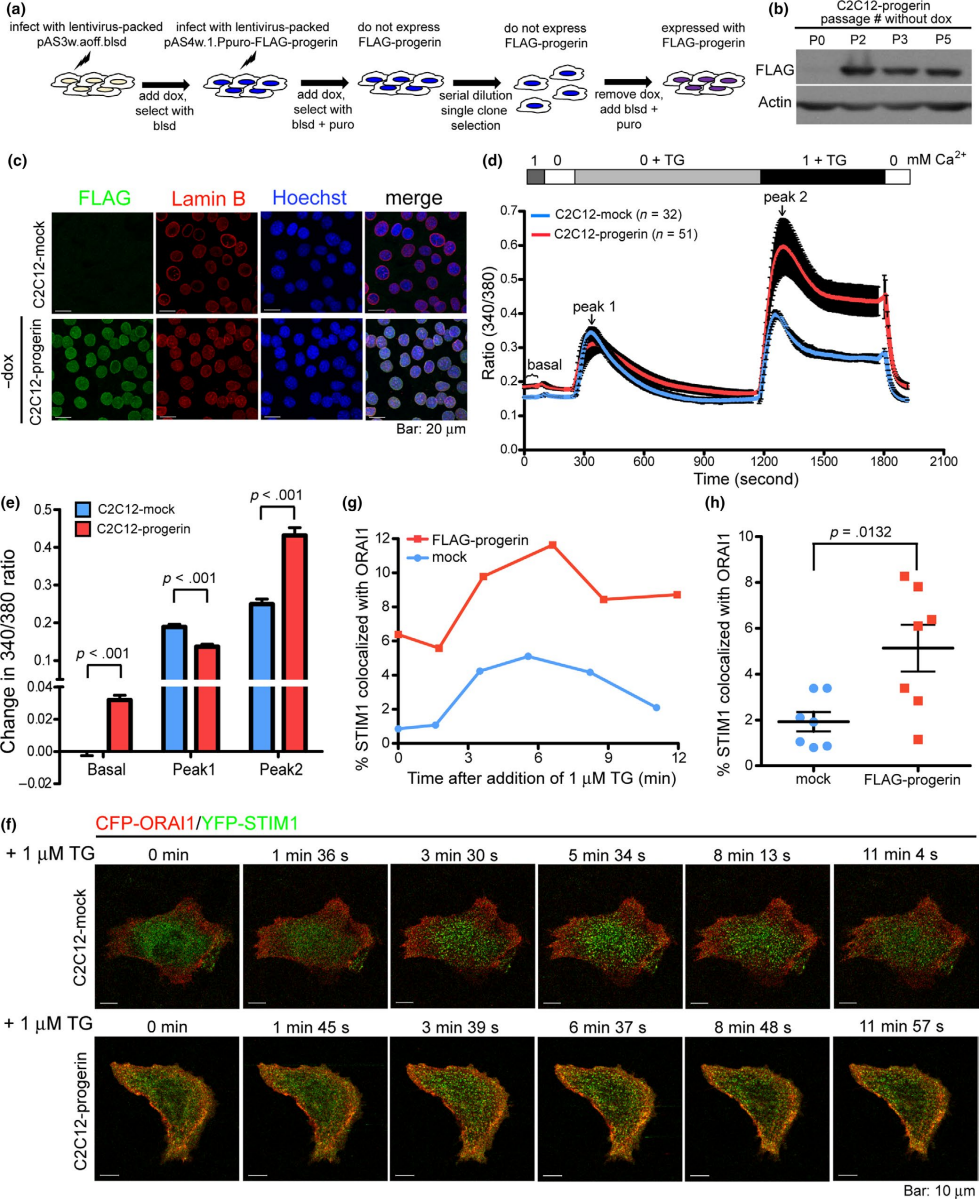
Enhanced store‐operated calcium entry in myoblasts expressing progerin. (a) Schematic illustration of the experimental procedures for the generation of C2C12 cell line inducibly (by doxycycline removal) expressed with progerin. Dox, doxycycline; blsd, blasticidin; puro, puromycin. (b) Western analysis for the expression of progerin (tagged with FLAG) before (i.e., P0) and after (P2, P3, and P5) removal of doxycycline. (c) Immunofluorescence staining for the expression of FLAG‐progerin (green) in the C2C12‐progerin stable cell line at passage 5 after removal of doxycycline. The immunofluorescence staining of FLAG in C2C12‐mock cells is shown for comparison. The NE was labeled by immunofluorescence staining of cell endogenous lamin B (red). The nuclei were counterstained with Hoechst 33,342 (blue). (d) Measurement of intracellular Ca^2+^ concentration in mock and progerin‐expressing C2C12 cells using a Fura‐2 AM calcium indicator. Images were recorded at a 5 s interval. (e) Mean differences in Fura‐2 AM emission ratios taken from (d). (f) Live confocal microscopy for the punctate formation of YFP‐STIM1 (green) and CFP‐ORAI1 (red) in mock and progerin‐expressing C2C12 cells. Images were recorded at the indicated time points before (i.e., 0 min) and after the treatment of 1 μM TG. (g) Percent co‐localization of YFP‐STIM1 with CFP‐ORAI1 as shown in (f). Images were processed using the Imaris software. (h) Percent co‐localization of YFP‐STIM1 with CFP‐ORAI1 in C2C12‐mock and C2C12‐progerin cells

The above results indicate that progerin interacts with Sln and Calnexin which modulate calcium uptake in the basal state (Bousette, Abbasi, Chis, & Gramolini, 2014). Therefore, we investigated the role of progerin expression in Ca^2+^ homeostasis. We failed in measuring Ca^2+^ signaling in flexor digitorum brevis (FDB) fibers isolated from *Lmna* ^−/−^ mice due to difficulties in culturing the tissue in vitro. Instead, we created a stable C2C12 myoblast cell line (i.e., C2C12‐progerin) capable of inducibly expressing progerin (tagged with FLAG) via doxycycline removal (Figure 5a,b). We employed an inducible system because we determined that progerin expression reduces C2C12 cell proliferation after several passages. The immunofluorescence imaging profile demonstrated the degree of homogeneity in progerin expression levels in C2C12‐progerin cells (Figure 5c). Progerin overexpression did not provoke the transcription of *Sln* (Figure S5a), perhaps due to the fact that *Sln* is specifically expressed in differentiated skeletal muscle (Maurya et al., 2018). We tried to differentiate C2C12 myoblasts into myotubes using 2% horse serum (HS); however, C2C12‐progerin cells failed to differentiate (Figure S5b). Nevertheless, we compared calcium efflux and influx in C2C12‐progerin and C2C12‐mock myoblasts using a fluorescent Ca^2+^ indicator, Fura‐2 AM. We recorded the fluorescence signal in the presence of 1 mM Ca^2+^ and then switched to a calcium‐free medium. The results indicate that the cytosolic Ca^2+^ level in C2C12‐progerin cells was higher than in the mock cells (Figure 5d, first 1 min record in the presence of 1 mM Ca^2+^; Figure 5e, basal). Under thapsigargin (TG, a SERCA inhibitor) treatment in the absence of extracellular Ca^2+^, the ER‐to‐cytoplasm Ca^2+^ efflux rate was ~ 28% lower in C2C12‐progerin cells (Figure 5d,e, peak 1). This result is similar to the findings presented by Rivera‐Torres et al., who demonstrated that the uptake and release of SR Ca^2+^ is blunted in *Zmpste24^−/−^* cardiomyocytes (Rivera‐Torres et al., 2016). Intriguingly, following the addition of extracellular Ca^2+^ in the presence of TG, the Ca^2+^ influx rate was markedly (~73%) higher in C2C12‐progerin cells than in the mock cells (Figure 5d, from 1,200 s; Figure 5e, peak 2).

Stromal interacting molecule 1 (STIM1) is an ER Ca^2+^ sensor that controls SOCE. STIM1 is able to sense a decrease in ER calcium content and activate plasma membrane ORAI channels to facilitate Ca^2+^ entry. STIM1 redistributes from a diffuse ER localization into puncta at the cell periphery upon ER store depletion (Roos et al., 2005). To determine whether the elevated calcium entry in progerin‐expressing C2C12 cells (Figure 5d,e) was correlated with an increase in the interaction between STIM1 and ORAI1, we co‐expressed CFP‐tagged ORAI1 and YFP‐tagged STIM1 in C2C12‐mock and C2C12‐progerin cells, and monitored their localization before and after the addition of 1 μM TG (Figure 5f,g). The results of immunofluorescence imaging revealed an increase in the co‐localization of ORAI1 and STIM1, even in the absence of TG treatment (Figure 5g,h). Collectively, these findings indicate that the basal cytosolic Ca^2+^ concentration and the mediation of SOCE by Ca^2+^ release‐activated Ca^2+^ (CRAC) channels are enhanced by the overexpression of progerin.

The expression of STIM1 has been shown to be induced by elevated intracellular Ca^2+^ levels (Luo et al., 2012). The enhanced muscle‐specific expression of STIM1 protein and a concomitant increase in sarcolemmal Ca^2+^ influx can alter the histological and biochemical characteristics of muscular dystrophy (Goonasekera et al., 2014). Our results showed that endogenous Stim1 was indeed elevated in C2C12‐progerin myoblasts, compared to the mock control (Figure S6a). Stim1 expression also appeared higher in P+M+ skeletal muscle than in the P+M− cohort (Figure S6b,c). This result indicates that the overexpression of progerin in muscle may elicit the expression of genes associated with calcium influx, and thereby dysregulate calcium homeostasis.

### 2.6 Increased ER stress in progerin‐expressing skeletal muscle

**Figure 6:**
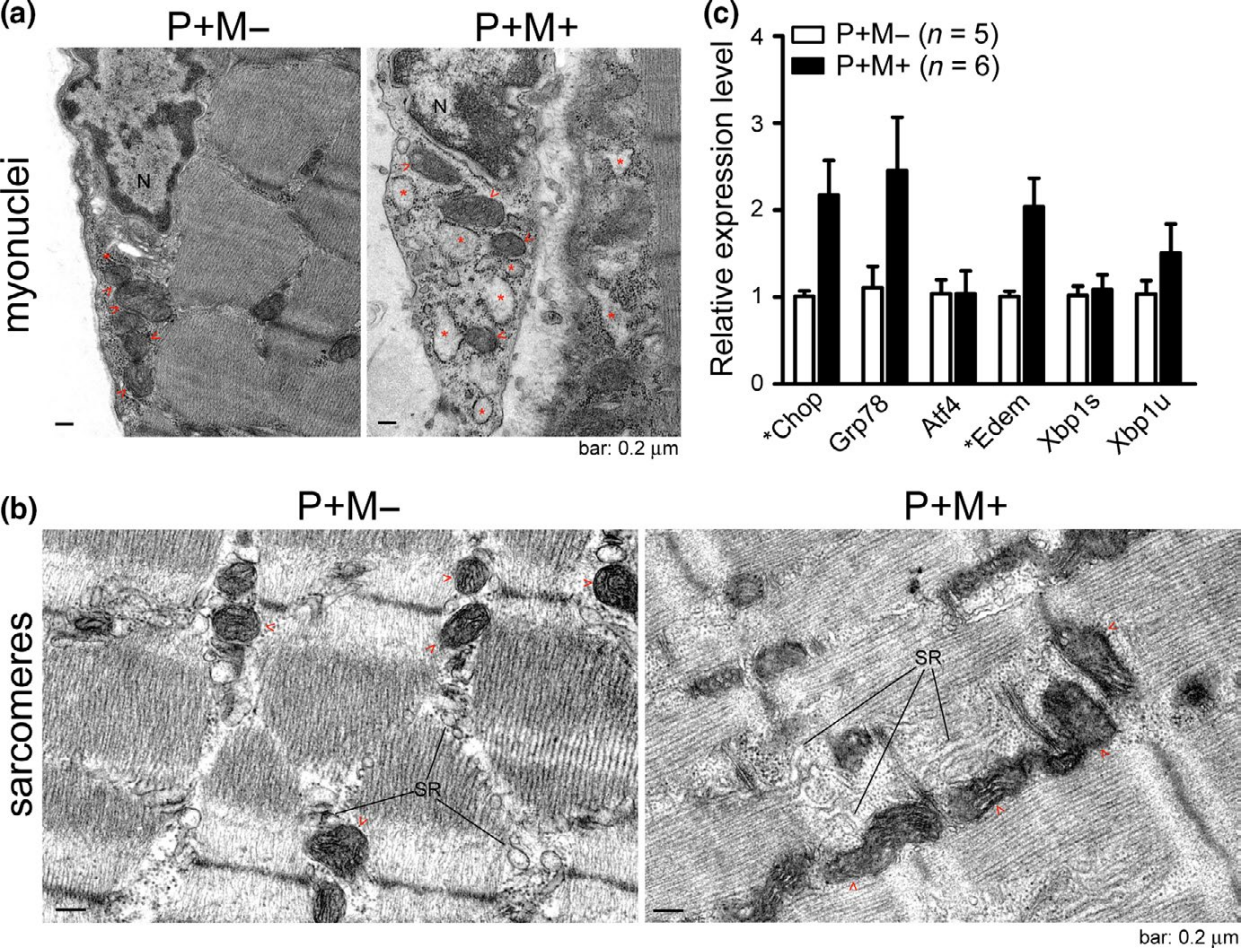
Increased endoplasmic reticulum (ER) stress and disrupted ER structure in the skeletal muscle expressing progerin. (a–b) TEM images of 61‐day‐old male P+M− and P+M+ gastrocnemius muscle recorded at 30,000× magnification. Dilated ER (denoted by red star sign), disorganized SR network and mitochondria (denoted by red arrow heads) with reduced cristae density were observed in P+M+ gastrocnemius muscle. N, nucleus; SR, sarcoplasmic reticulum. (c) qRT‐PCR for the expression of ER stress‐associated genes (Chop, p = .029; Edem, p = .020; Grp78, p = .068; Atf4, p = .9995; Xbp1s, p = .765) in the gastrocnemius and soleus muscle of 2‐month‐old P+M− and P+M+ mice. *p < .05, t test. All values are mean ± SEM. Xbp1s, spliced Xbp1; Xbp1u, unspliced Xbp1

We previously discovered that the structure of the ER is disrupted in HGPS skin fibroblasts (Chen et al., 2014). We therefore examined the ultrastructure of the ER and SR in progerin‐expressing skeletal muscle. The results of transmission electron microscopy (TEM) revealed that the ER lumen (denoted by red star sign) in the myonuclei of P+M+ mice was significantly dilated compared to that in P+M− mice (Figure 6a). We also observed a reduction in the density of the mitochondrial cristae (denoted by red arrow heads, Figure 6b).

Dilation of the ER lumen is a hallmark of ER stress (Oslowski & Urano, 2011). We then examined the expression of ER stress signals in skeletal muscle. The transcription of *Chop*, *Edem*, and *Grp78* in gastrocnemius and soleus muscles was indeed higher in P+M+ mice than in P+M− mice (Figure 6c). Elevated ER stress signaling was also observed at the protein level (Figure S7). These results indicate that the alterations to the ER/SR structure were accompanied by elevated ER stress levels in skeletal muscle expressing progerin.

## 3 DISCUSSION

The versatility of the nuclear lamins is closely associated with proteins embedded in the nuclear membrane which is contiguous with the ER. However, the functional link between nuclear lamins and the ER has not been fully elucidated (Subramanian & Meyer, 1997; Ungricht & Kutay, 2015). This study provides evidence of a molecular link between lamin A and ER/SR function as indicated by energy expenditure in skeletal muscle. The conditional overexpression of human progerin in mouse muscle is sufficient to cause muscle degeneration and elicit premature death (Figure 2). The ER lumen in myonuclei expressing progerin was significantly dilated (Figure 6a), which is similar to our previous observation of HGPS skin fibroblasts (Chen et al., 2014). Analysis at the molecular level revealed that progerin recruits a subset of calcium‐binding ER proteins, such as Sln and Calnexin, to the nuclear periphery and enhances SOCE in myoblasts (Figures 4 and 5). ER is the major Ca^2+^ store inside the cell, and Ca^2+^ cycling across the SR membrane is linked to thermogenesis and heat production. The interaction between progerin and Ca^2+^‐regulating proteins (e.g., Sln and Calnexin) provides a molecular explanation for the multitude of metabolic phenotypes (Figures 2, 3, 4), such as muscle weakness and malfunctioning lipid turnover in laminopathies (Liao et al., 2016; Worman, 2012). Lamin A aberrant splicing (the cause of HGPS) does indeed occur in the cells of healthy (i.e., non‐HGPS) individuals (Olive et al., 2010; Scaffidi & Misteli, 2006). Therefore, the progerin‐inducible knock‐in mouse model presented in this study could serve as a mammalian model by which to study the physiological role of aging elicited through progerin accumulation under a wild‐type *Lmna* genetic background.

The laminopathies caused by mutations in *LMNA* present abnormalities in postmitotic tissues, such as muscle, nerve, heart, and adipose tissues (Perovanovic & Hoffman, 2018). However, the molecular studies were performed mostly using cell models that can proliferate and undergo mitosis (Ho & Lammerding, 2012; Perovanovic & Hoffman, 2018). Due to the multifaceted functions of nuclear lamins in replicating and nonreplicating cells, it is not clear whether the confounded clinical phenotypes in laminopathies are caused by molecular damages during cell replication or postmitosis. In the P+M+ mouse model in this study, progerin expression was induced by *MCK‐Cre*, which has been shown to express only in postfusion myotubes (Bi et al., 2016). The body weight of P+M+ mice was higher than that of age‐matched *Lmna* ^−/−^ littermates (Figure 1e and S1f; Sullivan et al., 1999); however, their medial lifespans were similar (Figures 1f and 2d). These results suggest that the molecular lesions that lead to the mortality of P+M+ and *Lmna* ^−/−^ mice are probably cell cycle independent.

Ca^2+^ is a versatile intracellular messenger involved in a wide range of cellular and physiological processes, including transcriptional activation, cell cycle control, muscle contraction, and lactation (Mooren & Kinne, 1998). The NE which is continuous with the ER stores Ca^2+^ around the nucleus, thereby allowing efficient regulation of nuclear Ca^2+^ for gene transcription and cell cycle progression (Mauger, 2012). However, it remains unclear how the nuclear membrane couples with the ER for the influx of calcium. Sln is a regulator of SERCA activity in muscle and plays an important role in regulating muscle thermogenesis and metabolism (Bal et al., 2012; Pant et al., 2016). Calnexin and Calreticulin are both Ca^2+^‐binding molecular chaperons in the ER that assist folding and subunit assembly of Asn‐linked glycoproteins. Calnexin is a type I membrane protein, whereas Calreticulin is a soluble molecule residing within the ER lumen (Danilczyk, Cohen‐Doyle, & Williams, 2000). In this study, we observed that wild‐type lamin A can pull down Sln and Calnexin but not Calreticulin (Figure 4c). The interactions of Sln or Calnexin with progerin were more pronounced than their interactions with wild‐type lamin A (Figure 4). Furthermore, overexpression of progerin elicited the protein level of Stim1 (Figure S6). Based on the role of Sln, Calnexin, and Stim1 in buffering Ca^2+^ concentration in the ER, we hypothesized that the enhanced interaction between progerin and these ER proteins should affect calcium influx or efflux. Consistent with this hypothesis, we observed that the expression of progerin increased the basal Ca^2+^ concentration in the cytosol of myoblasts and enhanced Ca^2+^ uptake during SOCE (Figure 5d–h). These results indicate that the nuclear lamins may play a role in regulating Ca^2+^ homeostasis by binding to a subset of ER proteins including Sln and Calnexin. SOCE involves a variety of Ca^2+^‐dependent signaling pathways and multiple transcription factors associated with muscle growth, differentiation, and remodeling (Stiber & Rosenberg, 2011); therefore, the dysregulated influx of calcium in progerin‐expressing myoblasts may contribute to the maladaptive remodeling of muscle observed in HGPS patients (Merideth et al., 2008).

Researchers have linked the upregulation of *Sln* to a variety of muscle‐related diseases, including muscular dystrophy (Fajardo et al., 2018). In this study, we determined that *Sln* is significantly upregulated in the skeletal muscle that expresses mutant lamin A (Figures 1b and 3a). We also determined that the ablation of *Sln* shortened the lifespan of *Lmna* ^−/−^ mice (Figure 1f). Based on the role of lamin A in nuclear functions, such as DNA damage repair and transcriptional control, we surmise that the upregulation of *Sln* may be part of a global process promoting oxidative metabolism as a means of coping with compromised muscle function for the maintenance of energy expenditure. The overexpression of SLN increases fat oxidation (Maurya et al., 2018); therefore, it is possible that the reduction in fat mass observed in *Lmna* ^−/−^ and P+M+ mice (Figure 3c) is a consequence of increased energy expenditure in skeletal muscle.

It is worth noting that the NE is contiguous with the ER, and tubular ER networks present a large number of membrane contact sites with other organelles, such as the Golgi apparatus, mitochondria, endosomes, and exosomes. Moreover, the ER structure differs markedly across cell types. It is therefore to be expected that the mechanisms underlying the pleomorphic phenotypes in the laminopathies are only now emerging.

## 4 METHODS—EXPERIMENTAL PROCEDURES

### 4.1 Animals

The human progerin knock‐in mouse (Figure S1a) and *Sln* knockout mouse (Figure 1c) were constructed through the service of Transgenic Mouse Core at National Taiwan University. The animal experimental procedures were reviewed and approved by Institutional Animal Care and Use Committees (IACUC) of the National Health Research Institutes (NHRI) and Laboratory Animal Center, College of Medicine of National Taiwan University. Detailed protocols are provided in Supplementary Methods.

### 4.2 Cell culture

HEK293TN (LV900A‐1, System Biosciences, Palo Alto, CA, USA) and mouse myoblast C2C12 (CRL‐1772, ATCC, Manassas, VA, USA) cell lines were maintained in high glucose Dulbecco's Modified Eagle Medium (DMEM, Thermo Fisher Scientific, Waltham, MA, USA) containing 10% fetal bovine serum (FBS), 2 mM l‐glutamine and antibiotics. Normal and HGPS human skin fibroblasts were obtained from the National Institute of Aging (NIA) Aged Cell Repository distributed by the Coriell Institute. Cells were maintained in Minimum Essential Media (MEM, Thermo Fisher Scientific) containing 15% FBS and supplemented with 2 mM l‐glutamine, 1 mM sodium pyruvate, and antibiotics.

### 4.3 Antibodies

Expression of proteins were detected using antibodies against mouse lamin A/C (SAB4200236, Sigma‐Aldrich, St. Louis, MO, USA), human lamin A (MAB3540, Millipore, Burlington, MA, USA), Sarcolipin (ABT13, Millipore), β‐tubulin (ab179513, Abcam, Cambridge, UK), FLAG (F7425, Sigma‐Aldrich), HA (H6908, Sigma‐Aldrich), lamin B1 (ab133741, Abcam), lamin B (sc‐6217, Santa Cruz, Dallas, TX, USA), Calnexin (MAB3126, Millipore), Calreticulin (PA3‐900, Thermo Fisher Scientific), SERCA2 (4388, Cell Signaling, Danvers, MA, USA), β‐ACTIN (A5441, Sigma‐Aldrich), Grp78 (ab108613, Abcam), Chop (2891, Cell Signaling), eIF2α (2103, Cell Signaling), phospho‐eIF2α (3398, Cell Signaling), Atf4 (SC‐200, Santa Cruz), IRE1α (3294, Cell Signaling), phospho‐IRE1α (PA1‐16927, Thermo Fisher Scientific), and Gapdh (GTX100118, GeneTex, Hsinchu, Taiwan).

### 4.4 Generation of inducible progerin‐expressing C2C12 cell lines

To generate tetracycline‐controlled expression of progerin in C2C12 myoblasts, cells were first transduced with lentivirus packed with pAS3w.aOff.Pbsd (RNAi Core, Academia Sinica, Taiwan) using pPACKH1 HIV Lentivector Packaging Kit (LV500A‐1, System Biosciences) by following the manufacturer's protocol. After 2 weeks of selection with blasticidin, cells were transduced with lentivirus packed with a tetracycline‐controlled FLAG‐progerin expression sequence (Chen et al., 2014). C2C12 cells that were integrated with the targeting sequences were selected with puromycin at 1.0 μg/ml under the treatment of doxycycline (2 μg/ml).

### 4.5 Calcium imaging

The intracellular calcium concentration was determined by Fura‐2 AM calcium imaging as described by Alansary et al. (Alansary et al., 2014). In brief, cells were washed twice with calcium recording buffer (155 mM NaCl, 4.5 mM KCl, 10 mM Glucose, 5 mM HEPES, 2 mM MgCl_2_, 1 mM CaCl_2_, pH 7.4) and loaded with 1 μM Fura‐2 AM (Thermo Fisher Scientific) in calcium recording buffer at room temperature for 20 min. Cells were then washed three times with calcium recording buffer. The live calcium imaging experiments were performed using a Leica AF 6000 LX microscope system (Leica Microsystems). Images were acquired by exciting the cells at 340 nm and 380 nm. The quantified images were plotted as 340/380 ratios after background subtraction.

### 4.6 Statistical analysis

Data and statistical analyses were performed using Microsoft Excel and GraphPad Prism software. Survival curves were analyzed using log‐rank (Mantel–Cox) test, and Gehan–Breslow–Wilcoxon test. Data were analyzed using two‐tailed Student's *t* test as appropriate. Statistical methods that are not two‐tailed *t* test are denoted in the legends. *p*‐values below .05 were considered significant.

## CONFLICT OF INTEREST

The authors declare no conflict of interest.

## AUTHORS’ CONTRIBUTIONS

Y.H.C. designed the research and wrote the article; W.P.W., J.Y.W., W.H.L., M.C.H., and Y.C.T. performed the experiments; C.H.K., T.F.T., and Y.H.C. analyzed the data.
