# Supplementary material for: Progerin in muscle leads to thermogenic and metabolic defects via impaired calcium homeostasis
Source: Aging Cell. 2019 Dec 12;19(2):e13090. doi: 10.1111/acel.13090 (PMC6996945; doi:10.1111/acel.13090)
Supplement: Supplementary file 1 [file ACEL-19-e13090-s001.docx]

**Progerin in muscle leads to thermogenic and metabolic defects via impaired calcium homeostasis**

Wan-Ping Wang^1†^, Jing-Ya Wang^1†^, Wen-Hsin Lin^1^, Cheng-Heng Kao^2^, Ming-Chun Hung^1^, Yuan-Chi Teng^3^, Ting-Fen Tsai^3,4^, Ya-Hui Chi^1,5*^

^1^Institute of Biotechnology and Pharmaceutical Research, National Health Research Institutes, Zhunan, Miaoli County 35053, Taiwan; ^2^Center of General Education, Chang Gung University, Taoyuan 33302, Taiwan; ^3^Department of Life Sciences and Institute of Genome Sciences, National Yang-Ming University, Taipei 11221, Taiwan; ^4^Institute of Molecular and Genomic Medicine, National Health Research Institutes, Zhunan, Miaoli County 35053, Taiwan. ^5^Graduate Institute of Biomedical Sciences, China Medical University, Taiwan.

^†^These authors contributed equally to this work

^*^ Corresponding author

Ya-Hui Chi

35 Keyan Road, Zhunan, Miaoli County 35053, Taiwan

Ph: +886-37-206166ext35718; Fax: +886-37-586456

Email: [ychi@nhri.edu.tw](mailto:ychi@nhri.edu.tw)

**Supplementary Information**

**Supplementary Methods**

**Progerin knockin mice**

To knockin human progerin in the *Gt(ROSA)26Sor* locus of mice, the progerin complementary DNA (cDNA) containing Kozak and FLAG tag sequences at 5’ end (Chen et al., 2014) was amplified by PCR and inserted in NheI and NotI of pBigT vector (Addgene, 21270). The resulting construct which contains a PGK-neo-4×pA cassette, FLAG-progerin and two loxP sites was inserted in PacI/AscI site of pRosa26PAm1 vector (Addgene, 15036), in which CAG (CMV early enhancer/chicken beta actin) serves as promoter and the neo (neomycin) gene serves as a selection marker (Murtaugh, Stanger, Kwan, & Melton, 2003; Srinivas et al., 2001). The final targeting construct contained a 1.1 kb of 5′ homologous arm and 4.3 kb of 3’ homologous arm (Supplementary Fig. S1A). The constructed targeting vector was confirmed by sequencing, linearized by XhoI restriction digestion and electroporated into mouse embryonic stem (ES) cells. The ES cells were selected by G418 (160 μg/mL). The correctly targeted ES clones were verified by Southern blotting, and then injected into mouse blastocysts. The chimera mice (F0) were cross bred with C57BL/6JNarl mice, and germline transmission of F1 mice was verified by genomic DNA PCR using primers 5’-ATGGAGATGACCTGCTCCATCAC-3’ and 5’-TTACATGATGCTGCAGTTCTGGG-3’. To achieve muscle-specific progerin expression, mice which contain the progerin transgenic allele were cross bred with MCK-Cre mice [B6.FVB(129S4)-Tg(Ckmm-cre)5Khn/J, 006475] obtained from the Jackson Laboratory (Bi et al., 2016; Bruning et al., 1998). Genomic DNAs of mice which carry the MCK-Cre transgene were verified by PCR using primers 5’-GTGAAACAGCATTGCTGTCACTT-3’ and 5’-TAAGTCTGAACCCGGTCTGC-3’.

***Sln* and *Lmna* knockout mice**

The *Sln* knockout mice was constructed using the CRISPR-Cas9 double nicking technique through the service of Transgenic Mouse Core at National Taiwan University. The single guide RNAs (sgRNAs) which target exon 2 of *Sln* were designed based on the online website tools (<http://www.genome-engineering.org/crispr/>). All putative off-targets with less than 3 mismatches to the CRISPR sgRNAs were verified by Cas-OFFinder website tools (<http://www.rgenome.net/cas-offinder/>) and online website tools developed by Zhang’s lab (http://crispr.mit.edu/). The sgRNAs (5’sgRNA: 5’-GAAGATGGAGAGGTCTACTC-3’ and 3’sgRNA: 5’-TCACTCCCTGGAGTATAGCA-3’) were cloned in the PX330 vector (Cong et al., 2013). Messenger RNA of Cas9, 5’sgRNA and 3’sgRNA were prepared by in vitro transcription using mMESSAGE mMACHINE^®^ T7 ULTRA kit (Thermo Fisher Scientific, AM1345). Cas9 mRNA was further polyadenylated using poly(A) tailing kit (Thermo Fisher Scientific, AM1350). The in vitro transcribed RNAs were purified using ethanol precipitation, and were mixed and injected into the pronuclei of fertilized zygotes. Eighty injected zygotes were transferred into ampulla of the oviduct (20 embryos per oviduct) of pseudopregnant ICR females. Genomic DNAs of the F0 mice were extracted and purified using phenol:chloroform:isoamyl alcohol (25:24:1). DNA fragment deletion in the genome was verified by PCR (using primers 5’-GCACAAACCCTCTCCTTTTCCAT-3’ and 5’-TAGAAAACATGCCAAGGACCTAGT-3’) followed by sequencing. The founder mouse which was completed deleted for the coding sequence of *Sln* (Fig. 1C) was used for the subsequent experiments. The *Lmna* knockout mice were obtained from the Jackson Laboratory (B6.129S1(Cg)-Lmnatm1Stw/BkknJ, 009125) (Sullivan et al., 1999). All the mice were maintained under a 12-hour light/12-hour dark cycle with free access to water and standard mouse diet.

**Mouse behavior and metabolism**

The mouse behavior and metabolism experiments, including home cage, metabolic cage, grip strength, body composition, 2D X-ray, electrocardiography and surface body temperature, were performed by specialists in the Taiwan Mouse Clinic, Academia Sinica. All the experiments were executed by following the standard operating procedures approved by the Taiwan Mouse Clinic (http://tmc.sinica.edu.tw/sops.html). To ensure quality of the results, home cage, metabolic cage and grip strength analyses were performed prior to other tests. The mice were approximately 2 months old (45-75 days) at the time of testing for behavior and metabolism. We examined only male mice in the behavior and metabolism experiments, due to the fact that energy homeostasis in females is strongly associated with the level of estrogen, which might otherwise complicate the readout results (Mitchell et al., 2016; Xu & Lopez, 2018). For home cage analysis, a fully automated behavior analysis system (Clever Sys Inc., Reston, VA, USA) was used to study unconstrained mouse behaviors in a home cage. Life behaviors of mice were monitored for 24 hours in a safe and comfortable environment using 12:12 hour light-dark cycle with constant control of temperature(21±2 ^o^C) and humidity (40-70 %). The metabolism parameters were acquired by CLAMS-HC (Comprehensive Lab Animal Monitoring System for HOME CAGES, Columbus Instruments, Chicago, IL, USA) at 23 ^o^C using default setting. For grip strength, a MK-380CM/R grip strength meter (Muromachi Kikai Co., Ltd., Tokyo, Japan) was used to examine the forelimb grip strength of a mouse. The grip strength values were obtained from an average of 5 tests for each mouse. A Bruker Minispec LF50 TD (time-domain)-NMR Body Composition Analyzer (Bruker, Billerica, MA, USA) was used to measure body composition. The Body Composition Analyzer acquires and analyzes TD-NMR signals from all protons in the entire sample volume and can provide 3 components of interest: Fat, Free Body Fluid, and Lean Tissue values, allowing multiple measurements during the life of the animal. The 2D X-ray images were obtained using a Micro Computed Tomography (micro-CT) Imaging System (SkyScan 1076, Bruker). Electrocardiography of mice were obtained using an electrocardiograph system consists of four main parts: the Electrocardiograph PowerLab 8/30 (ADINSTRUMENTS, Australia), four amplifiers and subject cables, electrodes (MLA1204 Needle Electrodes), and notebook with Chart 5 software and Cardio Axis Program (ADINSTRUMENTS). A NEC F30S infrared thermal imaging was used to examine heat radiation on the body surface of mice. Heat radiation values were obtained during the daytime, and represent the average of 3 measurements obtained within a period of 1 min.

**Plasmids**

Complementary DNAs of human lamin A (Addgene, 17662) and progerin (Addgene, 17663) were obtained from Addgene, amplified by PCR, and cloned into pcDNA3 vector (Thermo Fisher Scientific) inserted with two FLAG tags at the N-terminus (i.e. pcDNA3-FLAG-lamin A and pcDNA3-FLAG-progerin). The expression vector of mouse Sln (i.e. pcDNA3-Sln-HA) was constructed by cloning mouse Sarcolipin (Sln) amplified by PCR using cDNA template obtained from *Lmna^-/-^* mouse femoris muscle into pcDNA3 vector inserted with two HA tags at the C-terminus. To construct lentivial infectious clone of progerin, cDNA of progerin with two FLAG tags at 5’ end was amplified by PCR, and cloned into a retroviral vector pAS4w.1.Ppuro (RNAi Core, Academia Sinica, Taiwan). The expression vectors of CFP-ORAI1 (Addgene, 19757) and YFP-STIM1 (Addgene, 19754) were obtained from Addgene (Prakriya et al., 2006).

**Transfection of human skin fibroblasts**

Normal and HGPS skin fibroblasts were transfected with the pcDNA3-Sln-HA expression plasmid via Lipofectamine^TM^ 2000 (Thermo Fisher Scientific, 11668019) in Minimum Essential Media (MEM) containing 2% FBS. After transfection for 4 hours, the medium was replaced with fresh MEM containing 15% FBS. Four days after transfection, the cells were fixed using 4 % paraformaldehyde in 1×PBS for immunofluorescence staining.

**Microarray analysis**

Total mRNAs were isolated from mouse muscle using the RNeasy mini kit (Qiagen, Germantown, MD, USA). Quality and integrity of the mRNAs were verified using Agilent 2100 Bioanalyzer (Agilent, Santa Clara, CA, USA). cDNAs were synthesized using the GeneChip™ WT PLUS Reagent Kit (Thermo Fisher Scientific), and was then hybridized to the Affymetrix Mouse Gene 2.0 ST array (Thermo Fisher Scientific). The microarray results were analyzed by Partek Pathway and Ingenuity® Pathway Analysis (IPA®) tools.

**Real-time quantitative PCR (qRT-PCR)**

Total mRNAs were isolated from mouse tissues using RNeasy mini kit (Qiagen) or TRIzol™ Reagent (Thermo Fisher Scientific). cDNAs were produced using SuperScript III Reverse Transcriptase (Thermo Fisher Scientific). qRT-PCR was carried out using Power SYBR Green master mix (Thermo Fisher Scientific). Gene expression levels were normalized to *Gapdh* or *Actb*. Primer sequences or commercial primers used for qPCR are listed as follows (5’ to 3’): *Lmna* (forward: GCTGACAAGGCTGCCGGTGG; reverse: CCAGGAGGTAGGAGCGGGTG), *Sln* (Qiagen, 330001 PPM27460F), *Ankrd1* (forward: CAGTGCAACACCAGATCCAT; reverse: ATGCCAAGGACAGAGAAGGA), *Mttp* (Qiagen, 330001 PPM24881A), *S100a4* (forward: GCTCCTTGAGCTCTGTCTTG; reverse: TCAGCACTTCCTCTCTCTTGG), *Cib2* (forward: CATGGACGATGGGACTCTTC; reverse: TTCACTGAAGAGCAGCTGGA), *Chop* (forward: CCTAGCTTGGCTGACAGAGG; reverse: CTGCTCCTTCTCCTTCATGC), *Grp78* (forward: TTCAGCCAATTATCAGCAAACTCT; reverse: TTTTCTGATGTATCCTCTTCACCAGT), *Atf4* (forward: GGGTTCTGTCTTCCACTCCA; reverse: AAGCAGCAGAGTCAGGCTTTC), *Edem* (forward: CTACCTGCGAAGAGGCCG; reverse: GTTCATGAGCTGCCCACTGA), *Xbp1s* (forward: GAGTCCGCAGCAGGTG; reverse: GTGTCAGAGTCCATGGGA), *Xbp1u* (forward: AAGAACACGCTTGGGAATGG; reverse: ATCCCCCTTGGCCTCCAC), *Gapdh* (Qiagen, QT01658692), *Actb* (Qiagen, QT00095242).

**Immunofluorescence staining and confocal microscopy**

Cells were fixed in 4% paraformaldehyde for 30 mins at room temperature and permeabilized with 0.5% Triton X-100 in phosphate buffered saline (PBS) for 10 mins. Cells were incubated with 1 % bovine serum albumin (BSA, Sigma-Aldrich, A9647) in PBS for 30 mins in order to block nonspecific binding. Primary antibodies were applied to cells, and incubated for 1.5 hours at room temperature. Fluorescent (Alexa-488, Alexa-568 or Alexa-633)-conjugated secondary antibodies (Thermo Fisher Scientific) were used for detection. Cell nuclei were counterstained with Hoechst 33342 (Thermo Fisher Scientific) and the slides were mounted with Prolong Gold antifade reagent (Thermo Fisher Scientific. Fluorescent images were recorded using a Leica TCS SP5 confocal microscope (Leica, Wetzlar, Germany). For live cell imaging, cells were incubated in a humidified chamber maintained at 37^o^C and supplied with 5% CO_2_. Images were processed using Imaris 7.2 software (Bitplane, Zurich, Switzerland).

**Co-immunoprecipitation and Immunoblotting**

The co-immunoprecipitation using DSP [Dithiobis (succinimidyl proprionate), Thermo Fisher Scientific] crosslinking were performed by following the protocol described by Lynes et al. (Lynes et al., 2013). In brief, HEK293TN cells seeded in 6-well plate were transfected with equal total amount of a mixture of vehicle plasmid (i.e. pcDNA3), the expression vectors of mouse Sln (pcDNA3-Sln-HA), human lamin A (pcDNA3-FLAG-lamin A), and/or progerin (pcDNA3-FLAG-progerin) . After 48 hours of transfection, cells were washed with PBS++ (phosphate-buffered saline containing 0.9 mM CaCl_2_ and 0.5 mM MgCl_2_) and incubated for 30 mins at room temperature with 2 mM DSP [Dithiobis (succinimidyl proprionate), Thermo Fisher, 22585] in PBS++ to crosslink interacting proteins. To quench the crosslinking reaction, cells were washed twice more and incubated in 10 mM NH_4_Cl in PBS++ for 10 mins. Then cells were washed once with PBS++ and harvested in CHAPS lysis buffer (1% CHAPS, 10 mM Tris pH 7.4, 150 mM NaCl, 1 mM EDTA) containing cOmplete protease inhibitors (Roche, 4693132001) and PhosSTOP (Roche, 4906845001). Cell supernatants were obtained by centrifuging the lysates at 800× g for 5 mins at 4 ̊C, and were incubated with monoclonal anti-HA (Sigma-Aldrich, A2095) or anti-FLAG agarose beads (Sigma-Aldrich, A2220) for 16 hours at 4 ^o^C. After three washes with CHAPS lysis buffer, the co-immunoprecipitated products were lysed in 1× SDS sample buffer containing β-mercaptoethanol. Lysates and immunoprecipitates were analyzed by SDS-PAGE, transferred to polyvinylidene fluoride (PVDF, Millipore) membranes, and blotted with primary antibodies. Corresponding alkaline phosphatase-conjugated secondary antibodies (Sigma-Aldrich) were added, and the blots were developed by chemiluminescence in accordance with the manufacturer’s protocol (PerkinElmer, Waltham, MA).

**Supplementary References**

Bi, P., Yue, F., Sato, Y., Wirbisky, S., Liu, W., Shan, T., . . . Kuang, S. (2016). Stage-specific effects of Notch activation during skeletal myogenesis. *Elife, 5*. doi:10.7554/eLife.17355

Bruning, J. C., Michael, M. D., Winnay, J. N., Hayashi, T., Horsch, D., Accili, D., . . . Kahn, C. R. (1998). A muscle-specific insulin receptor knockout exhibits features of the metabolic syndrome of NIDDM without altering glucose tolerance. *Mol Cell, 2*(5), 559-569.

Chen, Z. J., Wang, W. P., Chen, Y. C., Wang, J. Y., Lin, W. H., Tai, L. A., . . . Chi, Y. H. (2014). Dysregulated interactions between lamin A and SUN1 induce abnormalities in the nuclear envelope and endoplasmic reticulum in progeric laminopathies. *J Cell Sci, 127*(Pt 8), 1792-1804. doi:10.1242/jcs.139683

Cong, L., Ran, F. A., Cox, D., Lin, S., Barretto, R., Habib, N., . . . Zhang, F. (2013). Multiplex genome engineering using CRISPR/Cas systems. *Science, 339*(6121), 819-823. doi:10.1126/science.1231143

Lynes, E. M., Raturi, A., Shenkman, M., Ortiz Sandoval, C., Yap, M. C., Wu, J., . . . Simmen, T. (2013). Palmitoylation is the switch that assigns calnexin to quality control or ER Ca2+ signaling. *J Cell Sci, 126*(Pt 17), 3893-3903. doi:10.1242/jcs.125856

Mitchell, S. J., Madrigal-Matute, J., Scheibye-Knudsen, M., Fang, E., Aon, M., Gonzalez-Reyes, J. A., . . . de Cabo, R. (2016). Effects of Sex, Strain, and Energy Intake on Hallmarks of Aging in Mice. *Cell Metab, 23*(6), 1093-1112. doi:10.1016/j.cmet.2016.05.027

Murtaugh, L. C., Stanger, B. Z., Kwan, K. M., & Melton, D. A. (2003). Notch signaling controls multiple steps of pancreatic differentiation. *Proc Natl Acad Sci U S A, 100*(25), 14920-14925. doi:10.1073/pnas.2436557100

Prakriya, M., Feske, S., Gwack, Y., Srikanth, S., Rao, A., & Hogan, P. G. (2006). Orai1 is an essential pore subunit of the CRAC channel. *Nature, 443*(7108), 230-233. doi:10.1038/nature05122

Srinivas, S., Watanabe, T., Lin, C. S., William, C. M., Tanabe, Y., Jessell, T. M., & Costantini, F. (2001). Cre reporter strains produced by targeted insertion of EYFP and ECFP into the ROSA26 locus. *BMC Dev Biol, 1*, 4.

Sullivan, T., Escalante-Alcalde, D., Bhatt, H., Anver, M., Bhat, N., Nagashima, K., . . . Burke, B. (1999). Loss of A-type lamin expression compromises nuclear envelope integrity leading to muscular dystrophy. *J Cell Biol, 147*(5), 913-920.

Xu, Y., & Lopez, M. (2018). Central regulation of energy metabolism by estrogens. *Mol Metab, 15*, 104-115. doi:10.1016/j.molmet.2018.05.012

**Supplementary Figures and Legends**

**
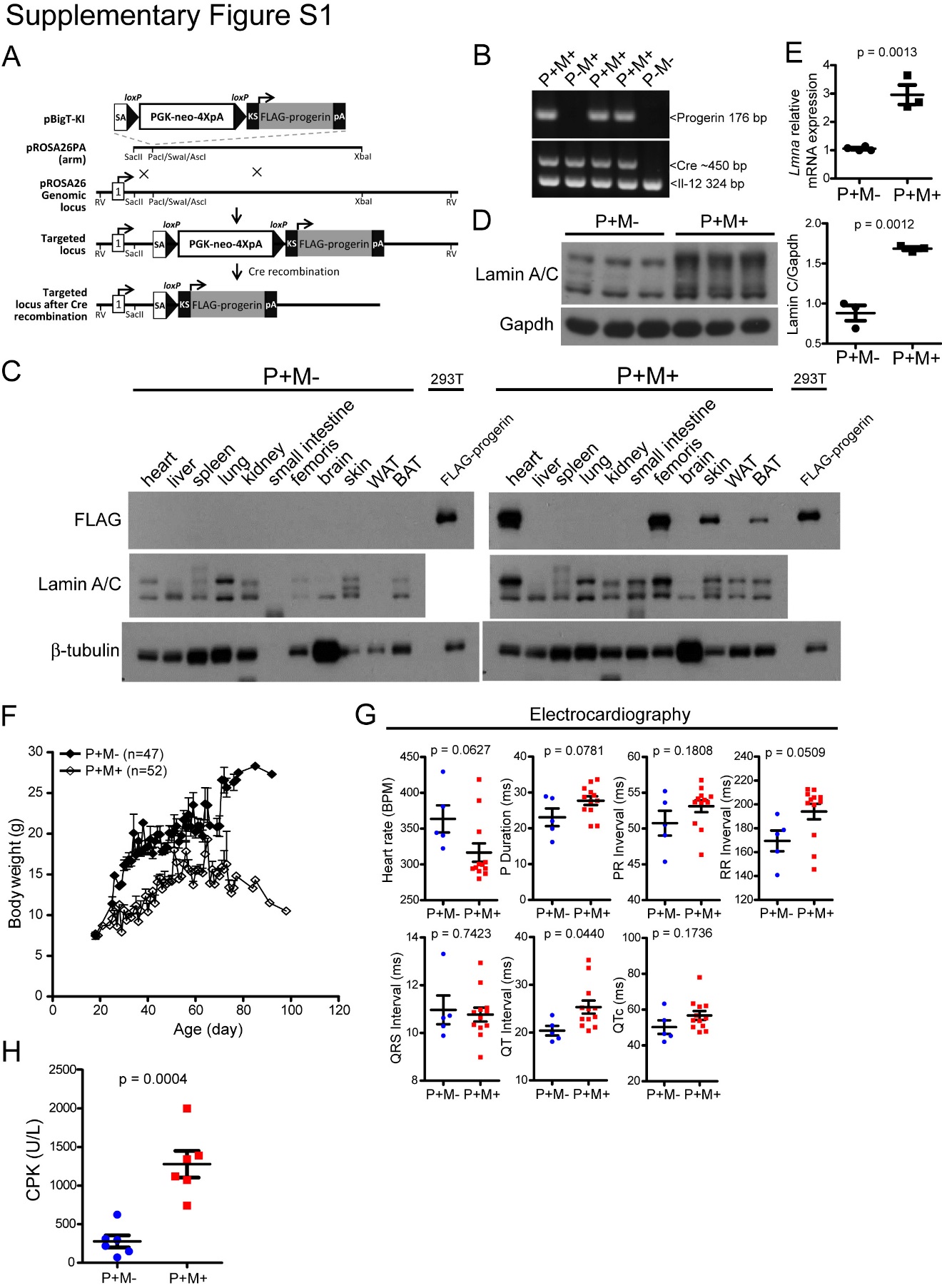
**

**Supplementary Figure S1. Generation of progerin-inducible expression in mice.** (A) Schematic representation of the targeting vector and the targeted ROSA26 locus. The targeting vector contains FLAG-progerin and a PGK-neo-4×pA cassette for selection. The PGK-neo-4×pA cassette was removed with the expression of Cre recombinase. (B) PCR analysis for the representative offsprings from heterozygous mating of *CAG-Progerin^+^* with *MCK-Cre^+^* mice. PCR of the knockin progerin generates a 176 bp fragment, and the knockin Cre generates a 450 bp fragment. PCR of the endogenous Il-2 (324 bp) serves as an internal positive control. The annotation “P+M+” indicate mice which harbor *CAG-Progerin^+^* and *MCK-Cre^+^* transgenes; the annotation “P-M-“ indicates mice which do not contain *CAG-Progerin* or *MCK-Cre* transgene. (C) Western analysis for the expression of endogenous lamin A/C and the knockin FLAG-progerin in various organs of 2-month-old male *CAG-Progerin^+^; MCK-Cre^+^* (P+M+) and *CAG-Progerin^+^* only (P+M-) mice. Cell lysates from HEK293TN (293T) cells overexpressed with FLAG-progerin was analyzed simultaneously as a positive control. Immunoblots of β-tubulin are presented as internal controls. (D) Western analysis for the expression of endogenous lamin A/C in the skeletal muscle of 2-month-old male P+M+ (n=3) and P+M- (n=3) mice. Expression of Gapdh is presented as an internal control. Quantification of the expression level of endogenous lamin C relative to Gapdh is shown at right of the immunoblot profiles. (E) Relative mRNA expression of *Lmna* in the skeletal muscle of 2-month-old male P+M+ (n=3) and P+M- (n=3) mice by qRT-PCR. (F) Growth curve in body weight of P+M+ and P+M- mice (male and female). (G) Electrocardiography (ECG) signals obtained from 2-month-old male P+M+ and P+M- mice. (H) Serum level of creatine phosphokinase (CPK) in 2-month-old male P+M+ (n=6) and P+M- (n=6) mice.

**
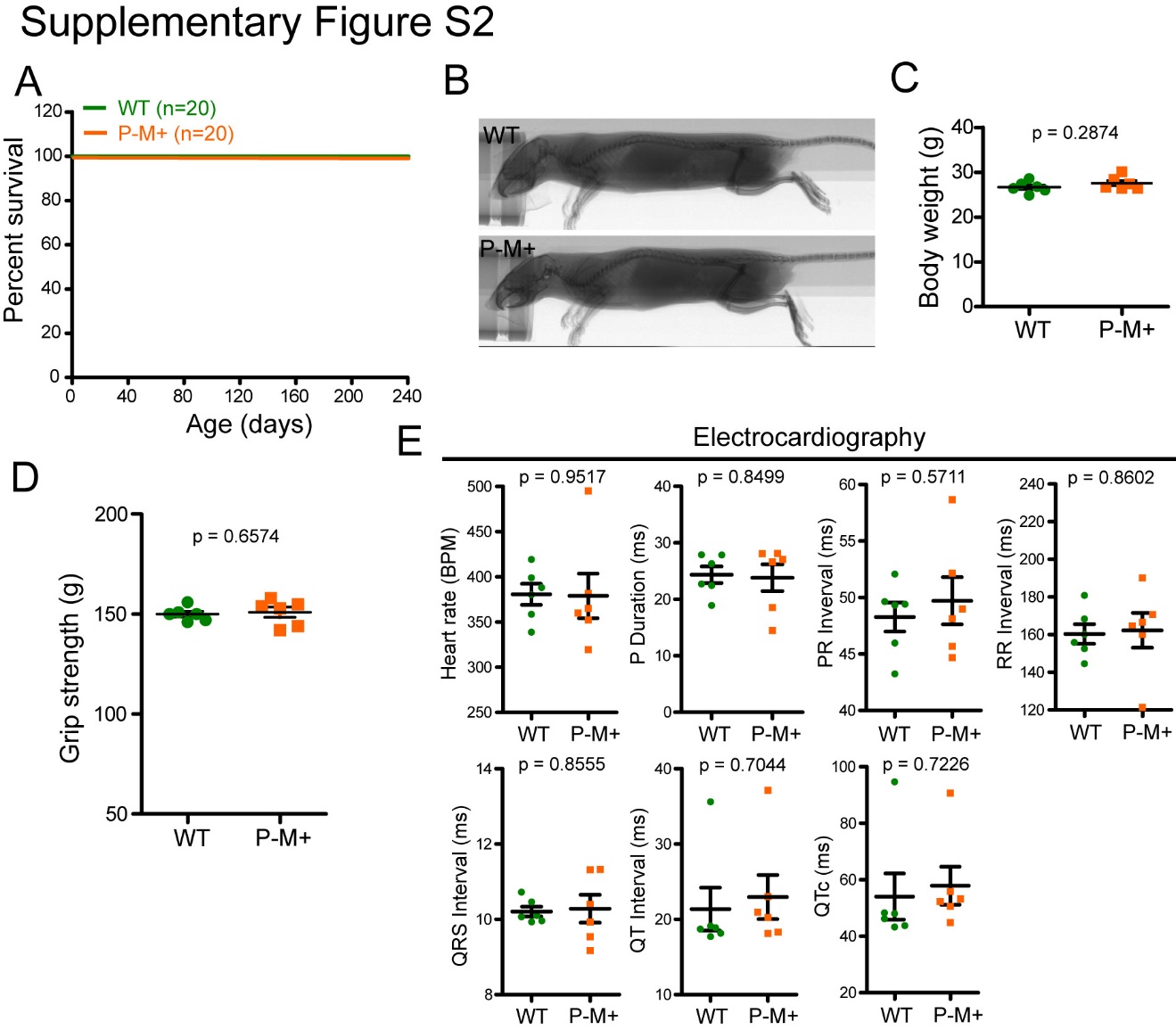
**

**Supplementary Figure S2. Behavior and electrocardiography of *MCK-Cre* transgenic mice.** (A) Kaplan-Meier survival curve of wild-type (WT) and *MCK-Cre^+^* only (P-M+) mice (male and female). All the examined mice can survive over 6-month old. (B) X-ray images of 3-month-old male WT and P-M+ mice. (C) Body weight of 3-month-old male WT (n=6) and P-M+ mice (n=6). (D) Grip strength of 3-month-old male WT (n=6) and P-M+ mice (n=6). (E) Electrocardiography (ECG) signals obtained from 3-month-old male WT (n=6) and P-M+ (n=6) mice.

**
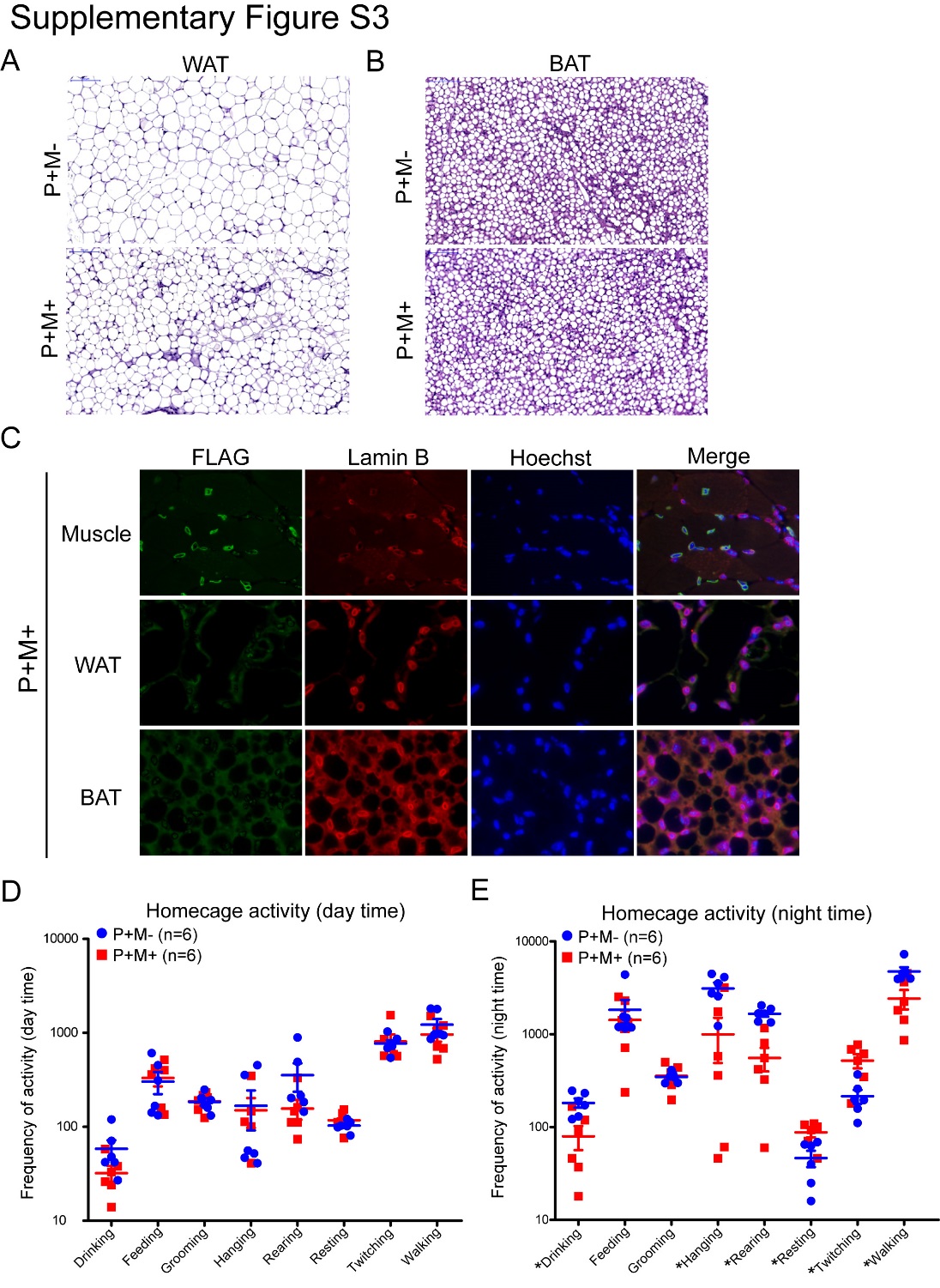
**

**Supplementary Figure S3. Histology of adipose tissues and homecage activity analysis.** (A-B) Histology analysis (200× magnification) of WAT (A) and BAT (B) using H&E staining paraffin sections from 2-month-old P+M- and P+M+ mice (male). (C) Immunofluorescence staining images (400× magnification) for the expression of FLAG-progerin (green) and endogenous lamin B (red) in the skeletal muscle, WAT and BAT of 2-month-old male P+M+ mice. Skeletal muscle, but not WAT or BAT, shows positive staining of FLAG. Cell nuclei were stained with Hoechst 33342 (blue). (D) Frequency of day time (7 am to 7 pm) and night time (7 pm to 7 am) activities (including drinking, feeding, grooming, hanging, rearing, resting, twitching, walking ) of 2-month-old male P+M- and P+M+ mice analyzed by the Clever Sys HomeCageScanTM3.0 software. *, p < 0.05.

**
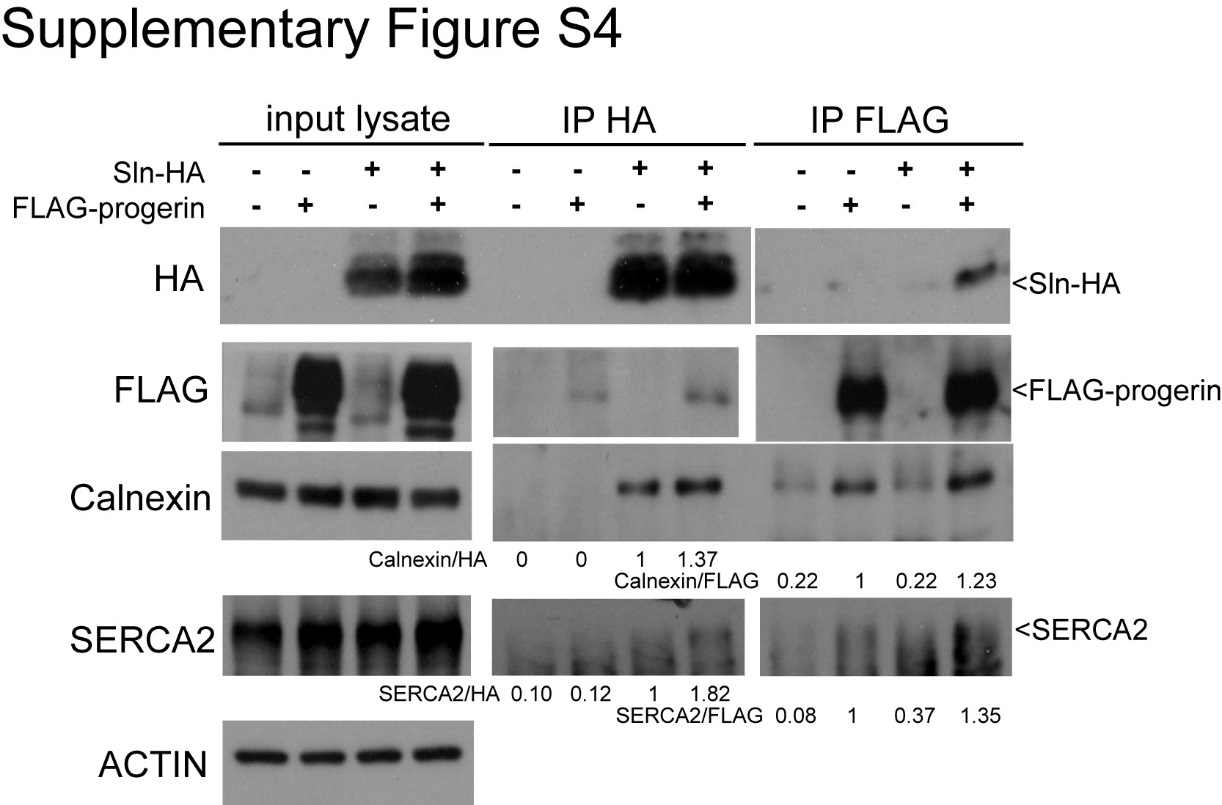
**

**Supplementary Figure S4.** **The interaction of progerin and ER proteins.** HEK293TN cells were co-transfected with Sln-HA and/or FLAG-progerin. Cell lysates were immunoprecipitated using mouse anti-HA and mouse anti-FLAG agarose, and the cell lysate input and co-immunoprecipitated products were analyzed by Western blot. The signals of the co-immunoprecipitated Calnexin and SERCA2 were quantified as shown below the immunostaining profiles.

**
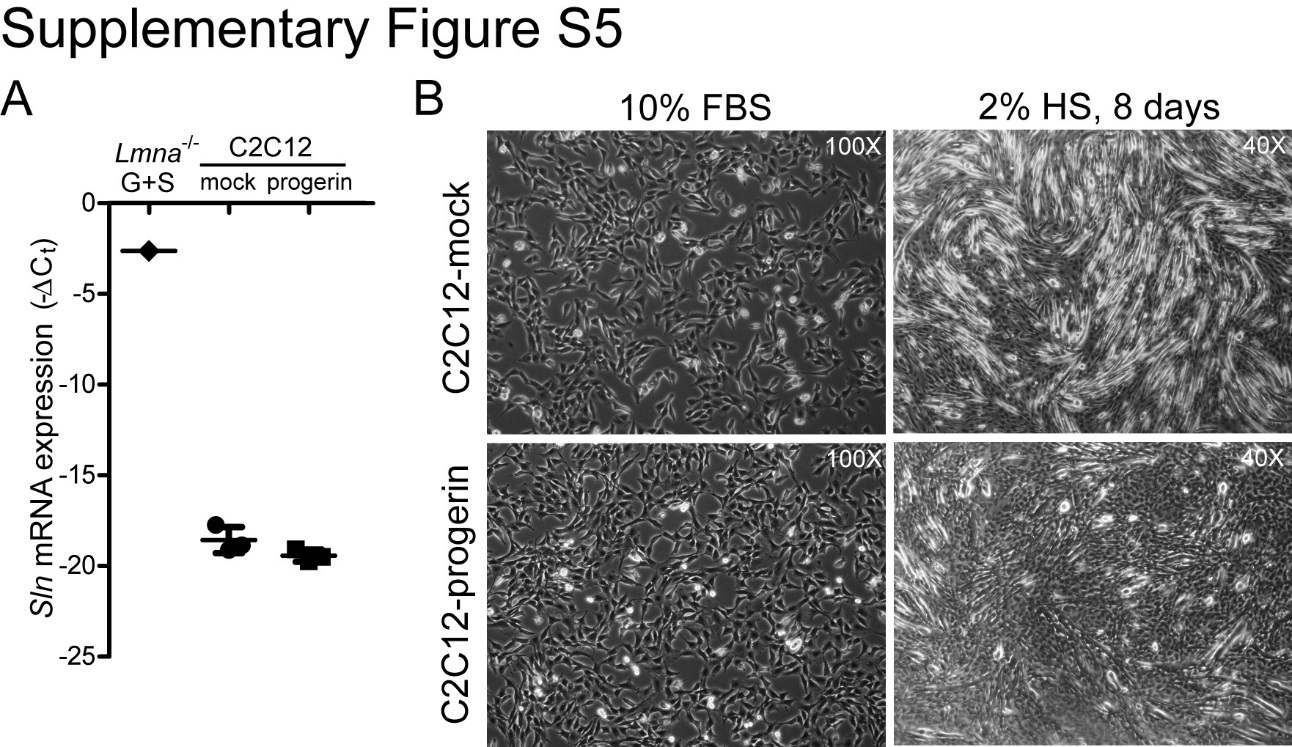
**

**Supplementary Figure S5. Expression of *Sln* in C2C12 myoblasts and effect of progerin expression on C2C12 myotube differentiation.** (A) Relative mRNA expression level of *Sln* in the gastrocnemius and soleus (G+S) muscle of an *Lmna^-/-^* mouse, C2C12-mock and C2C12-progerin myoblasts. The expression level of *Sln* in mock or progerin-overexpressing C2C12 myoblasts was about 15 log_2_ order less than in *Lmna^-/-^* skeletal muscle. (B) Morphology of C2C12-mock and C2C12-progerin myoblast cells cultured in 10% FBS (left panels, 100× magnification) and induced for myotube differentiation in 2% horse serum (HS) for 8 days (right panels, 40× magnification).

**
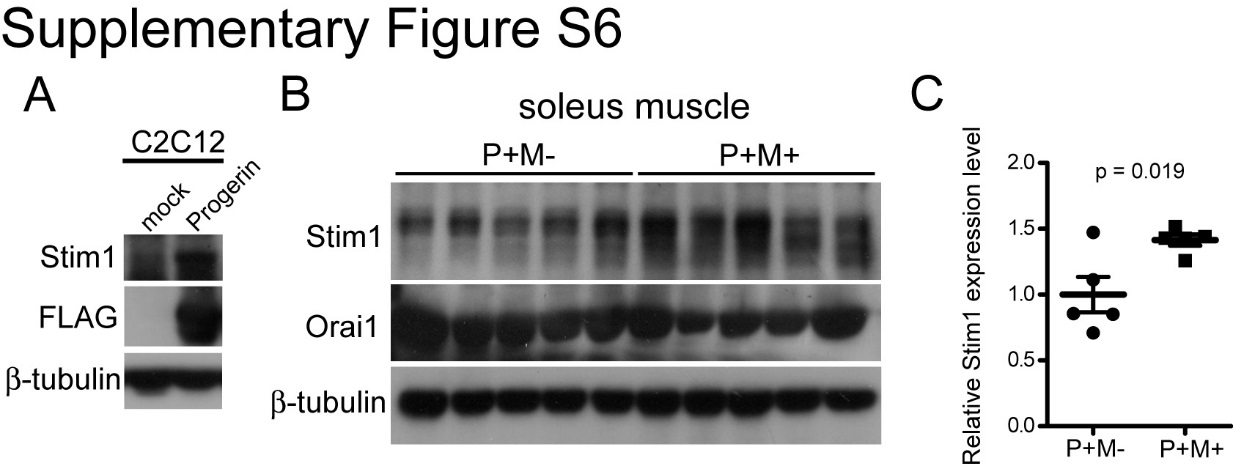
Supplementary Figure S6. Progerin overexpression upregulates Stim1 in muscle cells.** (A) Western analysis for the expression of endogenous Stim1 in C2C12-mock and C2C12-progerin stable clones. Immunoblot of β-tubulin is presented as an internal control. (B) Western analysis for the endogenous expression profile of Stim1 and Orai1 in the soleus muscle of P+M- and P+M+ mice (n=5 for each genotype). The expression level of Stim1 normalized to β-tubulin was quantified in (C).

**
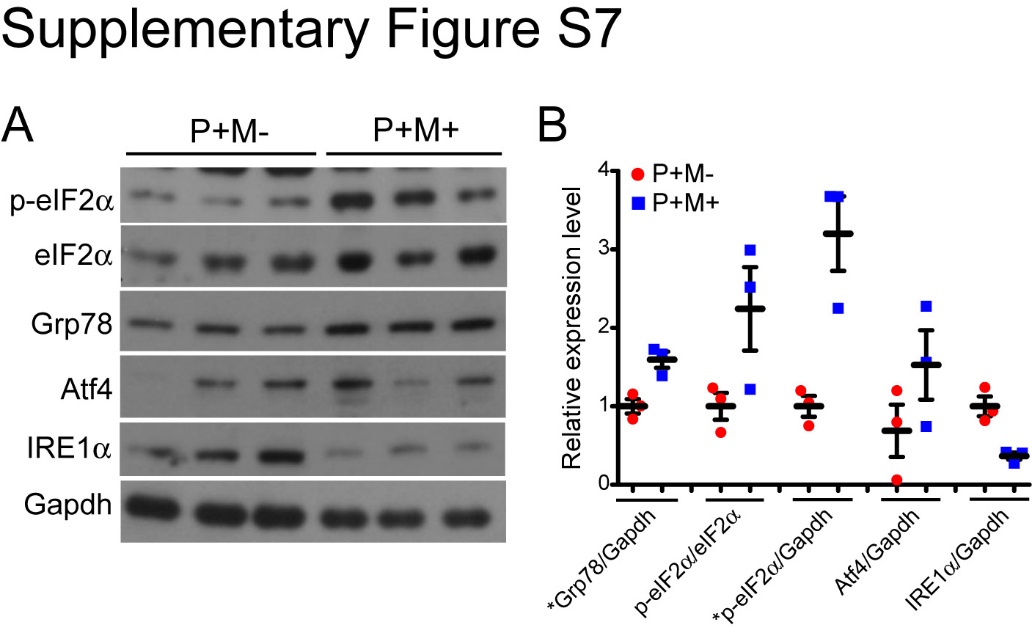
**

**Supplementary Figure S7.** Expression of ER stress-associated proteins in the skeletal muscle. (A) Western blot analysis for the protein expression of Grp78, eIF2α, phosphorylated eIF2α (p-eIF2α), Atf4 and IRE1α in the gastrocnemius and soleus muscle of 2-month-old male P+M- (n=3) and P+M+ (n=3) mice. We were not able to detect the expression of Chop or phospho-IRE1α in these tissues by Western analysis. The protein expression levels normalized to Gapdh or eIF2α are shown in (B). *, p < 0.05
